# Hexabromocyclododecane diastereomers in fish and suspended particulate matter from selected European waters—trend monitoring and environmental quality standard compliance

**DOI:** 10.1007/s11356-017-9469-4

**Published:** 2017-06-17

**Authors:** Heinz Rüdel, Josef Müller, Jens Nowak, Mathias Ricking, Roland Klein, Matthias Kotthoff

**Affiliations:** 1 Department Environmental Specimen Bank and Elemental Analysis, Fraunhofer Institute for Molecular Biology and Applied Ecology (Fraunhofer IME), 57392 Schmallenberg, Germany; 2 Department Environmental and Food Analysis, Fraunhofer Institute for Molecular Biology and Applied Ecology (Fraunhofer IME), 57392 Schmallenberg, Germany; 3 Department Earth Sciences, Anthropocene Research, Geochemistry, Freie Universität Berlin, 12249 Berlin, Germany; 4 Biogeography, University of Trier, 54286 Trier, Germany

**Keywords:** HBCD, Fish, Suspended particulate matter, Trend monitoring, Compliance monitoring, Environmental monitoring, Bream, Sole

## Abstract

The brominated flame retardant hexabromocyclododecane (HBCD) was monitored in fish and sediment (from one lake) or suspended particulate matter (SPM; from five rivers) at European freshwater sites to study the effects of reduction measures implemented by HBCD producers and users in recent years. Bream (*Abramis brama*) were sampled annually between 2007 and 2013 in the rivers Götaälv/SE, Rhône/FR, Western Scheldt/NL, Mersey/UK, and Tees/UK and in Lake Belau/DE. Sediment/SPM was taken every second year between 2008 and 2014. HBCD was analyzed by LC/MS/MS allowing the determination of the alpha-, beta-, and gamma-diastereomers. For most sites, a decrease in ∑HBCD was observed in fish (e.g., in the Rhône and Western Scheldt by about 80 and 60%, respectively, with significantly decreasing trends, *p* < 0.01). In the Rhône, HBCD also decreased in SPM. At the sampling site in the Tees which was impacted by a former HBCD point source, fish HBCD levels decreased only after a major flood event in 2013. While fish data indicate a decline in environmental HBCD concentrations at most sites with diffuse emissions, SPM data were less conclusive. The European environmental quality standard for HBCD in fish of 167 μg kg^−1^ wet weight was met by all fish samples in 2013.

**Electronic supplementary material:**

The online version of this article (doi:10.1007/s11356-017-9469-4) contains supplementary material, which is available to authorized users.

## Introduction

Hexabromocyclododecane (HBCD; CAS 25637-99-4 for mixture of isomers) is a brominated compound with several stereoisomers of which one, 1,2,5,6,9,10-HBCD (CAS 3194-55-6), is used as an additive flame retardant. It is applied, e.g., in extruded (XPS) and expanded (EPS) polystyrene foams used as thermal insulation in the building industry. HBCD is also used as flame retardant for instances in upholstery textiles and in high impact polystyrene (HIPS) utilized in electrical and electronic equipment and appliances. The technical product contains the diastereomers α-HBCD (CAS 134237-50-6), β-HBCD (CAS 134237-51-7), and γ-HBCD (CAS 134237-52-8). The diastereomers are chiral and occur as pairs of enantiomers. With a fraction of 70–95%, γ-HBCD is the main component of technical HBCD whereas α- and β-HBCD account for only 5–30% (EU 2008). Technical HBCD also contains traces of two further diastereomers (δ- and ε-HBCD, Heeb et al. 2005). Risk assessment and chemical properties of HBCD are summarized, e.g., by UNEP (2010) (risk profile), ECHA (2008, 2016), and EU (2008, 2011).

HBCD is listed under the Stockholm Convention on Persistent Organic Pollutants (POPs; UNEP 2016), and its production and usage is restricted to its application in XPS/EPS in buildings (Stockholm Convention Annex A effective since November 2014; in the European Union (EU) implemented by Commission Regulation (EU) 2016/293, EC 2016a). In the EU, HBCD is also regulated under the REACH regulation (EC 2006): due to its assessment as persistent, bioaccumulative, and toxic (PBT), HBCD was identified as a substance of very high concern (SVHC) in October 2008 (ECHA 2008) and included in Annex XIV for authorization under REACH in February 2011. Since August 2015, only two authorized uses of HBCD are allowed in Europe with authorizations expiring in August 2017: (1) the manufacturing of solid unexpanded flame-retarded pellets for EPS production and (2) the manufacturing of EPS articles from HBCD-containing pellets for usage in building applications (EC 2016b).

HBCD (as sum of α-, β-, and γ-HBCD) is also considered as a priority substance under the Water Framework Directive (WFD, EC 2000a). In the current version of the environmental quality standards (EQS) directive (2013/39/EU; EU 2013), an EQS for fish of 167 μg kg^−1^ wet weight (tissue not specified) is given which is intended to prevent secondary poisoning of predators. To achieve a good chemical status of surface waters in Europe, HBCD concentrations in fish have to comply with the EQS by end of 2018. Member states are also required to monitor trends in HBCD levels.

In recent years, several investigations reported on the presence of HBCD in environmental matrices (reviews: Covaci et al. 2006; Law et al. 2006, 2014). European monitoring data for fish are discussed in Rüdel et al. (2012). Studies from Europe covering sediments were performed, e.g., by Eljarrat et al. (2004), Stiehl et al. (2008), Bogdal et al. (2008), Harrad et al. (2009), and Hloušková et al. (2014). HBCD levels in fish and sediment varied and were especially high near potential sources like industrial plants and waste water treatment plants (WWTPs).

In sediments, γ-HBCD was typically the dominant diastereomer, thus reflecting the high γ-HBCD fraction in technical HBCD. In fish (and other biota), α-HBCD prevailed. This is probably related to the higher bioaccumulation efficiency of α-HBCD (possibly because of its higher water solubility; Esslinger et al. 2011) as well as the effective bio-isomerization of β- and γ-HBCD to α-HBCD in fish (for both, uptake via diet and from the water phase; Du et al. 2012, Zhang et al. 2014). Harrad et al. (2009) also detected low levels of δ-HBCD in some fish from English lakes and hypothesized that it was formed by bio-isomerization of other HBCD diastereomers. In laboratory studies, also, HBCD metabolites (e.g., tetrabromocyclododecene; Zhang et al. 2014) were identified. In environmental samples, however, such transformation products have only been reported in very few studies, e.g., by Hiebl and Vetter (2007) who detected pentabromocyclododecene in fish.

Even before regulations on HBCD were implemented, producers and users had started a product stewardship scheme for the responsible management of HBCD and other brominated flame retardants (Voluntary Emissions Control Action Programme, VECAP; www.vecap.info). Beginning in Great Britain in 2004, an emission control program was implemented in order to reduce potential environmental burdens from HBCD production in Europe (VECAP 2015). A recent evaluation of European data on HBCD emissions in 2014 revealed that since 2008 emissions to land during production have ceased completely and emissions to air and water have been reduced by 50 and 33%, respectively (VECAP 2015).

To evaluate, among others, the impact and the relevance of implemented emission reduction measures, an environmental monitoring project for HBCD was initiated in 2007 by the “HBCD Industry Working Group”, a sector group of the European Chemical Industry Council (CEFIC). The study was designed to investigate temporal and spatial trends of HBCD in environmental matrices at different sites across Europe in the period 2007–2014. The project focused on environmental compartments which are potential sinks for HBCD as identified under consideration of the physico-chemical properties and the life cycle of HBCD-containing products. Samplings covered fish (annually, 2007–2013) and suspended particulate matter (SPM; every second year between 2008 and 2014) at up to six different locations in Europe. According to Schubert et al. (2012), continuously sampled SPM can be used as alternative to grab samples of surface sediment. For the differential analysis, a diastereomer-specific analytical method (LC/MS/MS) was applied which allows the quantification of the three major compounds (α-, β-, and γ-HBCD; degradation products were not covered). Fish monitoring data for the period 2007–2010 have already been reported (Rüdel et al. 2012). The present publication extends the time series for fish and compares the data to HBCD concentrations in SPM from adjacent sites covering a similar period. Additionally, further European monitoring data for HBCD were researched to allow for a broader discussion of the following questions:

1. Do the new fish data confirm the findings of the first monitoring period (Rüdel et al. 2012)?
2. Are environmental levels of HBCD declining after initiation of emission control measures (restrictions on HBCD usage in Europe became effective only after the period monitored here)?
3. Are significant trends or concentration changes (comparing beginning/end of monitored period) detectable?
4. Are the investigated fish concentrations in compliance with the new EU WFD EQS?

## Materials and methods

### Sampling sites

Rüdel et al. (2012) and Nguetseng et al. (2015) already described the sampling sites covered in this study (site characteristics and anthropogenic pressures). Geo-coordinates are listed in the Electronic Supplementary Material (Table S1). Samplings of bream were performed in the period 2007–2013 at the following river sites: Götaälv/SE (no sampling 2009–2011), Mersey/UK (no sampling 2009–2011), Tees/UK, Western Scheldt/NL, and Rhône/FR as well as at Lake Belau/DE. Lake Belau was selected as a site with low anthropogenic impact.

### Sampling and sample preparation of fish

Sampling and sample treatment of fish (*Abramis brama*, bream; all sites; sole, *Solea solea*; only Western Scheldt) followed guidelines of the German Environmental Specimen Bank (ESB) and are described in Rüdel et al. (2012). If available, 15 fish per site were caught after the spawning season. The filets were dissected and combined for the preparation of annual pool samples. Samples were stored at temperatures <−150 °C and analyzed within 6 months after sampling. Bream had a trophic level (TL) of about 2.5–3.9 (Nguetseng et al. 2015; based on stable nitrogen isotope analysis) at the investigated sites. According to FishBase (Froese and Pauly 2016), bream mainly feed on benthic chironomids, crustaceans, mollusks, and plants resulting in a generic TL of 3.1 ± 0.1 (based on diet studies).

### Sampling and sample treatment of SPM and sediment

The SPM sampling followed the procedure described by Schulze et al. (2007) using stainless steel traps which were emptied every 3 months. At the river sites, SPM sampling campaigns were performed covering periods of 1 year each (from autumn to autumn, respectively; the sampling campaigns are designated by the year in which the major part of the sampling occurred). SPM was kept frozen at <−150 °C after sampling. After freeze-drying, the samples were prepared routinely as annual composite samples from equal amounts of the four 3-month periods. At Lake Belau, sediment was collected by core sampling (every second year in autumn/winter). For each sampling, 16 cores with an inner diameter of 4.5 cm were collected and frozen at <−150 °C directly after sampling. In the laboratory, the upper sediment layer of about 2 cm was cut with a stone saw from the frozen core, freeze-dried, and homogenized. The sediment core samples are designated by the year in which the major part of the sedimentation occurred. In April 2013, an additional grab sampling of surface sediment was performed in the Tees River upstream and downstream of the barrage near Stockton. Samples were stored at temperatures <−150 °C and analyzed within 6 months after sampling.

### Analysis

#### Materials

Standards (13C-labeled and non-labeled) of α-, β-, and γ-HBCD were purchased from Wellington Laboratories (via Campro Scientific, Berlin, Germany). Purities of the standards were >98%. All chemicals used were of HPLC or trace analysis grade. Glassware was treated at 250 °C for at least 12 h before each use with the exception of volume measuring devices (e.g., calibrated flasks) which were only rinsed with n-pentane and dried at room temperature before use. The preparation of HBCD standard solutions has already been described (Rüdel et al. 2012).

#### Fish sample preparation and analysis

The procedure is described in Rüdel et al. (2012). Briefly, samples of fish filet were homogenized after addition of acetone followed by extraction with n-pentane, and centrifugation. The extract was evaporated, re-dissolved, and purified by gel permeation chromatography (GPC). The solvent of the HBCD-containing GPC fraction was completely evaporated and the residue was re-dissolved in 100 μL acetonitrile for analysis. α-, β-, and γ-HBCD were quantified by liquid chromatography-mass spectrometry (LC/MS/MS) coupling a Waters 2695 HPLC system to a Micromass Quattro Micro triple quadrupole mass spectrometer (from Waters, Eschborn, Germany). For separation, a Phenomenex Gemini column (5 μm, C18, 150 mm × 3 mm; from Phenomenex, Aschaffenburg, Germany) was used applying the conditions given in Rüdel et al. (2012). Measurements were performed via negative electrospray ionization in the multiple reaction monitoring mode (collision gas: argon). Usually, two to four replicates of the annual pool samples were analyzed. Fish concentrations are either reported as wet weight (ww) or lipid weight (lw). Data are given either diastereomer-specific or as sum of the three quantified HBCD diastereomers (ΣHBCD).

#### SPM/sediment sample preparation and analysis

After mixing with sodium sulfate, freeze-dried sediment and SPM samples were extracted with dichloromethane by accelerated solvent extraction (ASE). Twenty-two-milliliter ASE cells were completely filled with 5 g of the SPM sample (exactly weighed) and about 18 g of sodium sulfate. Then, 100 μL of IS solution (100 ng mL^−1^ of each ^13^C-labeled analyte in acetonitrile) were added. Extractions were performed with dichloromethane at 15 MPa and 100 °C (heat time 5 min, static time 10 min). ASE extracts were evaporated and re-dissolved in n-hexane followed by a column clean-up (1 g silica gel with 1.5% water). After rinsing with 9 mL of a solution of 15% dichloromethane in n-hexane, HBCD-containing fractions were eluted by passing 10 mL of 30% dichloromethane in n-hexane through the silica gel columns. After drying the eluate with a nitrogen evaporator, residues were dissolved in 100 μL acetonitrile and analyzed by LC/MS/MS. Measurement conditions were similar to the fish analysis settings. Annual pool samples were typically analyzed two to four times and data are either reported on a dry weight (dw) basis or after normalization to the total organic carbon (TOC) content.

#### Calibrations

For daily calibrations, at least six concentration levels were applied which were adapted to the HBCD levels in each set of samples (range, 0.5–350 μg L^−1^ plus blank for fish; for SPM, an additional calibration solution of 500 μg L^**−1**^ was applied).

#### Fat determination in fish filet

For total lipid determinations of fish, a gravimetrical procedure was applied (Smedes 1999). TOC determination: TOC was determined by dry combustion following the standard EN 15936 (2012) or the previous standard ISO 10694 (1995).

#### Statistical evaluations

Arithmetic mean values were calculated from measured concentrations even in cases where these were in the range between the limit of quantification (LOQ) and the limit of detection (LOD). In the tables, these values are shown in brackets. Concentrations of ΣHBCD in fish and SPM are also reported after normalization to fat and TOC, respectively. Annual arithmetic mean values of individual HBCD diastereomers and ΣHBCD were calculated for replicates or for individual fish (Lake Belau bream 2008, Western Scheldt sole 2007). Time series with four or more successive annual samplings were analyzed for possible trends by applying the two-sided non-parametric Mann-Kendall test. Significance levels of the respective trends were calculated with a Microsoft Excel application developed by Salmi et al. (2002). Furthermore, trend analysis was performed using the Microsoft Excel-based software tool LOESS-Trend, Version 1.1 (developer: J. Wellmitz, German Environment Agency). The program fits a locally weighted scatterplot smoother (LOESS) with a fixed window width of 7 years through the annual HBCD levels and tests the significance of linear and non-linear trend components by an analysis of variance (ANOVA) following the procedure of Fryer and Nicholson (1999).

Biota-sediment accumulation factors or biota-suspended solids accumulation factors (BSAFs/BSSAFs) were calculated based on the lipid-normalized fish muscle HBCD concentrations (Tables S3 and S5, Electronic Supplementary Material; assumption: the lipid-normalized concentration of the filet is representative for the whole fish; see e.g., EC 2014) and the TOC-normalized sediment/SPM data (Table S8) according to Burkhard et al. (2012): *c*_fish_ (μg kg^−1^ lipid weight) / *c*_solid_ (μg kg^−1^ TOC), with: *c*_fish_ = ΣHBCD concentration in fish and *c*_solid_ = ΣHBCD concentration in sediment/SPM.

### Validation and quality assurance

For method validation, the guidelines SANCO 3029 and 825 (EC 2000b, 2004) were applied. Accordingly, fortification experiments were conducted at least at the concentration of the LOQ and a tenfold higher concentration. As sample matrix, bream and SPM samples from sites with low HBCD levels were chosen. In procedural blanks, levels of each of the three HBCD diastereomers were <0.1 μg kg^−1^ ww for fish and <0.1 μg kg^−1^ dw for SPM, respectively. Calibration functions were recorded for each measurement series and yielded for the linear regression coefficients of determination *r* ^2^ of >0.999 for each HBCD diastereomer. For fish, LOQs of 0.1 μg kg^**−1**^ ww and, for SPM/sediment, LOQs of 1.0 μg kg^**−1**^ dw were confirmed. Recoveries of the fortification tests (between 70 and 110%) and relative standard deviations (RSD), as measure for analytical precision, were sufficient (<20%). The RSD for fish was 7–19% for the LOQ level and 2–9% for the 10 * LOQ level. For SPM/sediment, the RSD was 3–4% for the LOQ level and 4–7% for the 10 * LOQ level. Concentration data were not adjusted for recoveries. Based on the method validation, both the fish and SPM methods were accredited under ISO/IEC 17025 (2005).

Subsamples of appropriate bream and SPM samples were applied as laboratory reference materials. These were run with each set of samples. The determined mean concentrations ± standard deviations for the fish material were 9.6 ± 1.1 μg kg^**−1**^ α-HBCD, 0.1 ± <0.1 μg kg^**−1**^ β-HBCD, and 0.8 ± 0.1 μg kg^**−1**^ γ-HBCD (ww data; *n* = 18). For the SPM material, the respective concentrations were 4.1 ± 1.8 μg kg^**−1**^ α-HBCD, 1.0 ± 0.4 μg kg^**−1**^ β-HBCD, and 28.0 ± 7.8 μg kg^**−1**^ γ-HBCD (dw data; *n* = 9). The reproducibilities of the methods were assessed as sufficient.

## Results and discussion

### Characterization of fish

Detailed information on sampling and the respective biometric data of the fish was given in Rüdel et al. (2012) and Nguetseng et al. (2015) and is summarized in the Electronic Supplementary Material (“Fish Yield and Biometric Data” section and Table S1).

At the Western Scheldt, sole (*Solea solea*) were sampled in addition to bream. Sole are well adapted to brackish estuarine waters. The age of the sole was around 3 years and thus younger than bream sampled at the same site. However, according to Bromley (2003), sole mature at an age of 3 years, and the sole caught for this study were thus considered appropriate.

The sampling at the Tees River (UK) in 2013 yielded bream with a significantly lower weight and size. This is probably related to a major flood event in that region in the previous year (winter 2012/2013) which removed most of the sediment fraction <2 mm from the sampling area (see discussion below). Since bream mainly feed on sediment, this probably reduced the food availability causing decreased fish abundances as well as reduced fish sizes due to migration (see discussion below). On the other hand, fish health parameters such as fat content and condition factor (see Electronic Supplementary Material, “Fish Yield and Biometric Data” section) of the bream from 2013 were comparable to those of the previous years.

The fat fraction of the fish was usually measured for pooled samples (only occasionally, individual fish were analyzed). At Lake Belau, the fat content of bream pools varied between 0.9% (2007) and 3.1% (2009). At the other sites the fat content were in the ranges of 2.5–3.6% (Götaälv), 1.9–3.4% (Tees), 0.8–3.6% (Rhône), and 1.9–2.9% (Mersey). At the Western Scheldt, fat in bream samples ranged between 2.4% (2010) and 5.0% (2011) and between 0.6% (2007) and 2.3 (2011) in sole.

Despite small deviations from the intended fish characteristics, the pools were assumed to be representative for the populations at the respective sites. Due to the lipophilic properties of HBCD (the n-octanol/water partition coefficient as log *K*_OW_ is 5.625; EU 2008), an accumulation in fat is expected. To cover for differences in fat between years, normalization to the lipid content was performed (e.g., Law et al. 2014 and EC 2014).

### HBCD diastereomer patterns and concentrations in fish

HBCD concentrations of bream and sole are listed in Tables S2, S3, S4, and S5 (Electronic Supplementary Material; ww and lw data). It has to be emphasized that not all possibly relevant HBCD diastereomers were covered in this study. Harrad et al. (2009) reported on the presence of δ-HBCD in fish with levels up to 11% of total ΣHBCDs although in most fish, δ-HBCD levels were below the LOQ. It is thus assumed that the general pattern would not change significantly even if δ-HBCD was included. Likewise, no HBCD transformation products like pentabromocyclododecene were analyzed here because they were detected only in a few studies up to now (e.g., Hiebl and Vetter 2007).

In most fish samples, α-HBCD was the dominant diastereomer (Table 1). This finding is in agreement with published data (e.g., Covaci et al. 2006, Gerecke et al. 2003, Harrad et al. 2009, Hloušková et al. 2013, Miège et al. 2012). An exception was Lake Belau where in most years, γ-HBCD was the dominant diastereomer in bream tissue. Only in 2009, the α-HBCD fraction was larger than that of γ-HBCD. A similar diastereomer pattern was observed for the sole from the Western Scheldt with γ-HBCD fractions of up to 74% (Table 1). So far, no conclusive explanation for the altered pattern is available. Comparable patterns were reported, e.g., by Harrad et al. (2009) for individual fish from English lakes.

**Table 1:** HBCD diastereomer pattern in annual pool samples of muscle tissue from bream (*Abramis brama*) and sole (*Solea solea,* only Western Scheldt) sampled in different European fresh waters between 2007 and 2013. Fractions of HBCD diastereomers in %

Site	Mean (range) of diastereomer fraction			Remark
	α-HBCD	β-HBCD	γ-HBCD	
Lake Belau	33% (20–70%)	10% (8–12%)	56% (22–68%)	Highest fraction of α-HBCD in 2009
Götaälv	70% (34–95%)	5% (1–10%)	25% (4–55%)	Highest fraction of α-HBCD in 2013
Tees	97% (95–99%)	1% (1–2%)	2% (1–3%)	–
Rhône	84% (64–91%)	2% (1–5%)	14% (9–32%)	Lowest fraction of α-HBCD in 2011
Mersey	86% (83–91%)	4% (2–5%)	10% (6–13%)	–
Western Scheldt (bream)	82% (70–91%)	3% (1–5%)	15% (8–26%)	–
Western Scheldt (sole)	31% (20–56%)	11% (5–13%)	59% (33–74%)	High fraction of γ-HBCD in most years (exception: α-HBCD dominated in 2010)

Interestingly, the largest α-HBCD fractions (95–97%; Table 1) were detected in those bream samples that showed the highest overall HBCD concentrations (i.e., from the River Tees) whereas the low ∑HBCD concentrations of Lake Belau bream and Western Scheldt sole go hand in hand with high γ-HBCD fractions. However, for bream at the Western Scheldt and from other sites, α-HBCD remained the predominant diastereomer also in years with low ΣHBCD levels (e.g., Götaälv 2007/2008, Rhône 2010–2013). Concentrations of β-HBCD in fish were always low.

**Fig. 1:**
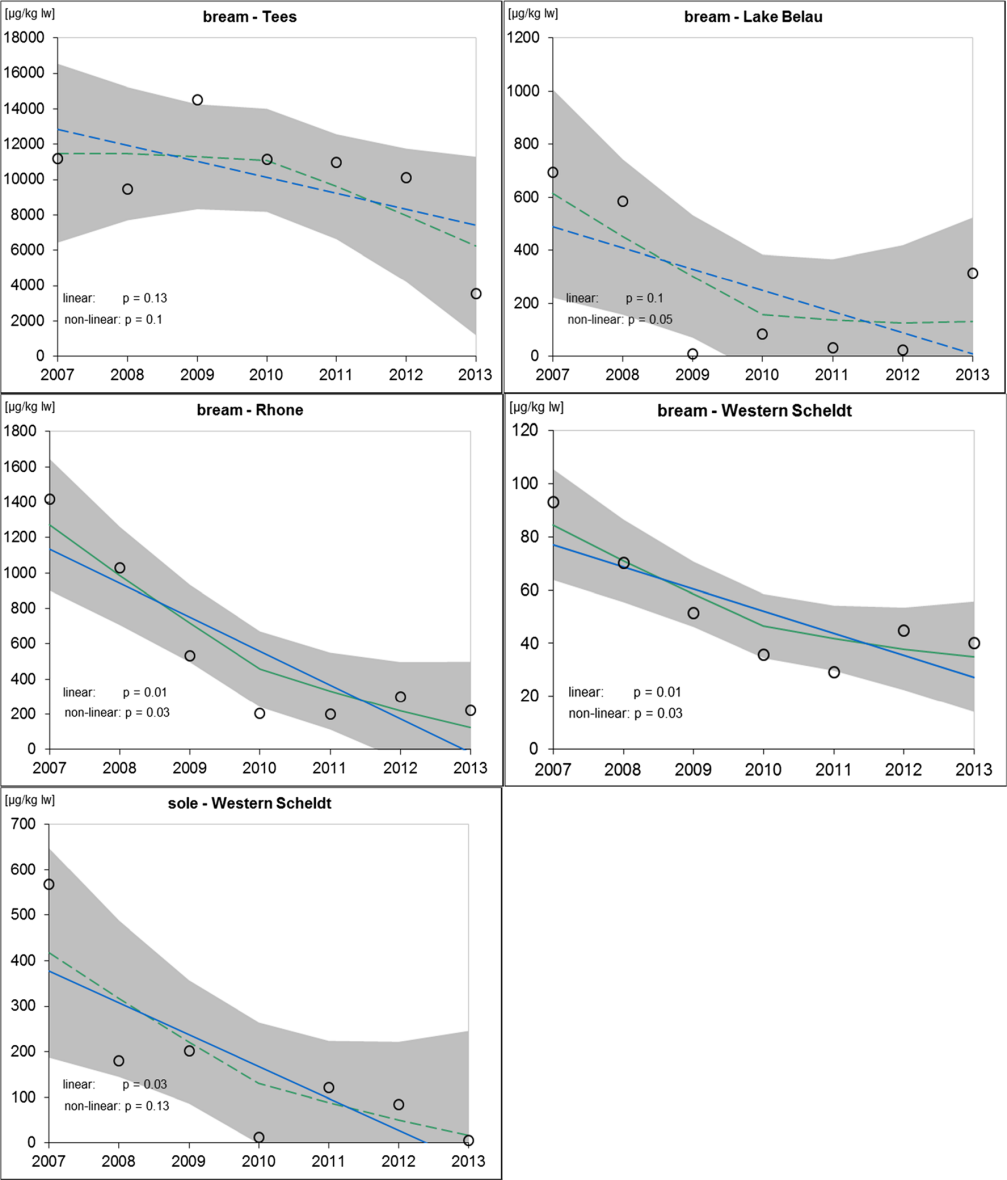
Temporal trends of ΣHBCD (sum of α-, β-, and γ-HBCD) concentrations in bream and sole muscle tissue (μg kg^−1^; lipid weight-based; mean data as given in Table S1, Electronic Supplementary Material). The *lines* show the linear regression and the LOESS smoother (*solid* for significant linear or non-linear trends, *broken* for not significant). *Shaded areas* represent the 95% confidence intervals of the LOESS function

The lipid weight-based ΣHBCD concentrations in fish are shown in Fig. 1 (annual ΣHBCD data and trend lines). Concentration data for the three diastereomers are displayed in Fig. S1 (Electronic Supplementary Material).

Bream from the Tees River were highly contaminated with levels around 11,000 μg kg^−1^ lw ΣHBCD in the period 2007 to 2012. An extremely high value of 14,500 μg kg^−1^ lw ΣHBCD was detected in 2009 while a sharp decline to 3540 μg kg^−1^ lw ΣHBCD was observed in 2013. In 2013, bream were smaller and lighter compared to previous years (Table S1, Electronic Supplementary Material). This may be related to massive flood events in winter 2012/2013: On the one hand, the floods may have altered the local bream population by shifting individual bream or even the entire bream population downstream. The highly contaminated bream could have been carried away from the sampling site and less contaminated smaller (but not necessarily younger) individuals from upstream sites could have taken their place. On the other hand, the floods may have washed a large fraction of the (probably HBCD-loaded) sediment away. A sampling in early 2013 revealed that some parts of the river bed were completely depleted of fine sediment (see below). This may have reduced the uptake of HBCD by bream during feeding at the sediment.

At the other three continuously sampled sites, decreasing HBCD levels were detected in the course of the study. At Lake Belau, ΣHBCD concentrations decreased from 695 μg kg^−1^ lw in 2007 to 315 μg kg^−1^ lw in 2013. The lowest levels were found in 2009 (11 μg kg^−1^ lw) and 2012 (24 μg kg^−1^ lw). The relatively high ΣHBCD levels of Lake Belau bream in some years (as compared to fish from, e.g., Götaälv River; see below) are nevertheless surprising since this site is characterized by low anthropogenic impacts.

In the Rhône, HBCD decreased by about 84% from 1417 μg kg^−1^ lw in 2007 to 222 μg kg^−1^ lw in 2013 with the lowest levels around 200 μg kg^−1^ found in 2010/2011.

For bream from the Western Scheldt, a 57% reduction of ΣHBCD was observed during the study period (2007: 93 μg kg^−1^ lw; 2013: 40 μg kg^−1^ lw) with the lowest concentration detected in 2011 (29 μg kg^−1^ lw). Clearly higher initial ΣHBCD concentrations and a stronger decrease were detected for Western Scheldt sole, i.e., from 568 μg kg^−1^ lw in 2007 to 12 μg kg^−1^ lw in 2010 and to levels below the limit of quantification (<10 μg kg^−1^ lw) in 2013 (measured value 5.6 μg kg^−1^ lw; total decrease 2007–2013: 99%). However, in 2011 and 2012, ΣHBCD levels in sole were relatively high with 120 and 85 μg kg^**−1**^ lw, respectively (Table S5, Electronic Supplementary Material). Currently, it is not clear why HBCD concentrations in sole muscle tissue were so much higher in 2007 and decreased faster than those in bream from the same site. Sole are in closer contact to the sediment than bream because they live burrowed in sandy and muddy grounds most of the time while bream stay in the free water. Their exposure via the surrounding media may thus be different as well as their capacity to bio-transform and degrade HBCD diastereomers. Since both species feed on worms, mollusks, and small crustaceans in the sediment or at the sediment/water interphase, the exposure via food should be quite similar (both represent a similar trophic level). However, bream in the Scheldt are caught near the river banks while sole are caught also in the midstream area where the sediment is more influenced by remobilization and deposition. Sediment and feed organisms from different sites of the stream bed may well have different HBCD levels which can eventually lead to different HBCD exposures of the fish.

At the river sites Mersey (UK) and Götaälv (SE), the sampling was continued in 2012 and 2013 after a gap of 4 years (sampled before only in the years 2007 and 2008). For bream from the Mersey, a significant decrease from the previous high levels was observed (about 50% decrease from 2007/2008 to 2012/2013) but levels were still high in 2013 (1730 μg kg^−1^ lw) compared to bream from the Rhône and Western Scheldt. The picture is less clear for the Götaälv. The ΣHBCD levels in bream muscle ranged around 40 and 80 μg kg^−1^ lw in 2007/2008 and 2013. In 2012, however, a high concentration of 307 μg kg^−1^ lw was measured which cannot be explained so far. Biometric data give no indication of significant differences between the fish caught in 2012 and 2013 (Table S1). Beside the increase in ∑HBCD levels in 2012, a shift in diastereomer pattern occurred from about 70–95% α-HBCD in 2007/2008/2013 to only about 35% in 2012. This shift may reflect different exposure conditions of the Götaälv fish in 2012.

### Statistical evaluation of temporal changes of HBCD levels in fish

The results of the statistical trend analysis based on lipid weight are presented in Fig. 1 (data: Table S3, Electronic Supplementary Material).

No significant trend was identified for HBCD levels in bream from the Tees River in UK when trend analysis was based on lipid weight (though ΣHBCD levels decreased sharply 2013; Fig. 1). However, when using the wet weight data, a significant decrease in ΣHBCD can be detected (*p* = 0.02; annual decrease between 2007 and 2013: 42 μg kg^−1^ ww) (Table S2, Electronic Supplementary Material). The decrease in HBCD between the years 2007–2009 on the one hand and 2010–2013 on the other is probably related to decreases in the fat content of the fish (2007–2009: 2.7–3.3 vs. 1.9–2.4% in 2010–2013). A reason for this change could not be identified. The biometric data for 2010–2012 were comparable to those for 2007–2009. Only in 2013, the fish were smaller and lighter.

For bream from Lake Belau, no significant decreasing trend was detected for ΣHBCD or any diastereomer because inter-year variations were high. In 2013, the HBCD concentrations in fish were higher than in the previous years but lower than at the beginning of the time series (about 45% of the ΣHBCD levels in 2007).

For the Rhône site, trend analysis revealed a significant linear decreasing trend for ΣHBCD (*p* = 0.01; annual decrease between 2007 and 2013, 192 μg kg^−1^ lw). The non-parametric Mann-Kendall test indicated significant trends for concentrations of α-HBCD (*p* < 0.05) and ΣHBCD (*p* < 0.1).

HBCD concentrations in Scheldt bream decreased significantly between 2007 and 2013 (linear trend for ΣHBCD: *p* = 0.01; annual decrease 8.3 μg kg^−1^ lw; Mann-Kendall test for α-HBCD and ΣHBCD: *p* < 0.1). Significant decreasing trends were also detected for sole from the Western Scheldt (linear trend for ΣHBCD: *p* = 0.03, annual decrease 70 μg kg^−1^ lw; Mann-Kendall test for ΣHBCD and γ-HBCD: *p* < 0.01) with measured HBCD concentrations below the LOQ/LOD in 2013.

For bream from the Mersey, no significant differences in the ΣHBCD were detected between consecutive years (2007/2008, respectively, 2012/2013) whereas significant differences were observed when comparing the combined mean ΣHBCD levels for 2007/2008 and 2012/2013 (Student’s *t* test, *p* < 0.01). However, since the years 2009–2011 were not covered in the study, it is not possible to decide whether these changes reflect a clear trend.

At the Götaälv, no significant differences were found in the HBCD burdens of bream caught in 2007, 2008, and 2013, whereas the combined mean value of these years differs significantly from the level in 2012 (Student’s *t* test, *p* < 0.01) which also displayed a different diastereomer pattern (see above).

To summarize, there is evidence that environmental HBCD levels at sites with originally high burdens (mainly influenced by local emissions such as WWTPs but excluding the legacy burden at Tees River) have decreased significantly. At sites with lower HBCD levels that stem probably from diffuse emissions (e.g., via atmospheric deposition; Okonski et al. 2014), the picture is less clear. For the future, the EU WFD requires trend monitoring for HBCD diastereomers in river basins. This will allow a broader analysis of temporal HBCD concentration data for European waters.

### Comparison of HBCD levels in fish with data reported in the literature

In the following, only more recent reports are considered that have been published after 2012 (for comparisons with earlier studies refer to Rüdel et al. 2012).

Two studies also covered sampling regions which are part of the current study. Miège et al. (2012) reported HBCD diastereomer levels of Rhône fish including bream (*A. brama*) sampled upstream and downstream of the city of Lyon between August 2008 and January 2009. The mean ΣHBCD concentrations in muscle tissue of bream were 202 ± 271 μg kg^−1^ dw and 46 ± 62 μg kg^−1^ ww, respectively (*n* = 9; data converted with an average fish tissue water content of 77% as given by Miège et al. 2012). These values are comparable to those presented in the current study for bream caught in the Rhône near Arles (about 250 km downstream of Lyon) in 2008 (i.e., ΣHBCD levels of 30 μg kg^−1^ ww). In a trend study covering several German freshwater sites, Fliedner et al. (2016) analyzed α-, β-, and γ-HBCD diastereomers in archived bream filet from the German ESB. Fish sampling and sample treatment were comparable since the current study also applied procedures of the German ESB. α-HBCD was the dominant diastereomer in the ESB study and accounted for 71–97% of the ΣHBCD concentrations. The lowest levels were detected in bream from Lake Belau which was also covered in the current study: for 2013, Fliedner et al. (2016) reported 0.17 μg kg^−1^ ΣHBCD in bream from Lake Belau which is clearly lower compared to that in the present study (4.1 μg kg^−1^). ΣHBCD levels of up to 45.6 μg kg^−1^ ww were found in the Saar and the lower Rhine. Trend analyses revealed decreasing HBCD levels between the mid-1990s and 2014 in the upper and lower Rhine (and at the upper Danube for a shorter time series) while concentrations increased in the Elbe, Saar, and middle Rhine (Fliedner et al. 2016).

In the north Italian lake Lago Maggiore, Poma et al. (2014) detected highly variable HBCD levels in muscle tissue of shad (*Alosa agone)* and whitefish (*Coregonus lavaretus*) sampled in 2011, i.e., between 13 and 574 μg kg^**−1**^ lw in shad and 64–792 μg kg^**−1**^ lw in whitefish. The higher concentrations are comparable to the levels detected in fish from Lake Belau in the present study in 2007/2008. Zacs et al. (2014) and Svihlikova et al. (2015) analyzed HBCD in fish samples from Latvian and Czech rivers. Baltic salmon from Latvia had ΣHBCD concentrations in the range of 0.39–3.82 μg kg^−1^ ww (Zacs et al. 2014) with α-HBCD as predominant diastereomer. These levels were comparable with those detected in bream from the Götaälv in the present study. In filet of fish from the Czech river section of the Elbe, ΣHBCD was in the range 3.15–1211 μg kg^−1^ lw with the highest amounts of ΣHBCD quantified in fish from a site downstream a factory producing polystyrene (Svihlikova et al. 2015). Again, α-HBCD was the dominating diastereomer. The higher levels reported by Svihlikova et al. (2015) are in the range found in Rhône fish in 2007/2008 in the present study.

Comparatively low ΣHBCD levels are reported by Vorkamp et al. (2014) for muscle tissue of perch collected in 2012 from Danish freshwaters. Concentrations ranged between about 0.01 and 0.02 μg kg^−1^ ww and were thus lower compared to those in other monitoring studies including the present one. Vorkamp et al. (2014) hypothesized that these low HBCD fish levels might be related to a lower exposure in Danish waters or to different biological factors (e.g., lower lipid contents of fish).

Altogether, the HBCD concentrations presented here are in accordance with published data for other freshwater fish species and/or sites in Europe with comparable anthropogenic burdens. Trend analyses reveal decreasing levels or a leveling off at most of the investigated sites in the last years. Since the uses of HBCD have recently been restricted (see “Introduction” section), it is expected that environmental inputs will continue to decline (see, e.g., VECAP 2015) and in its wake also the HBCD levels in fish (Fig. 1).

### EQS compliance of fish

In the present study, the HBCD levels of fish muscle samples were below the biota EQS of 167 μg kg^−1^ ww (EU 2013) at all sites and in all years except for bream from the Tees sampled between 2007 and 2012 where wet weight levels of about 200–400 μg kg^−1^ ΣHBCD were quantified.

The majority of HBCD fish monitoring studies quantified HBCD in the filets of relative large fish. However, since the biota EQS (EU 2013, EU 2011) was derived for the protection of predators (e.g., carnivore fish, otters, or herons) from secondary poisoning, the more appropriate sample matrix would be (smaller) whole fish picked as prey. If filet is used instead, the risk toward predators may be underestimated (EC 2014). According to data from laboratory bioconcentration tests the HBCD concentration ratio between whole fish and edible parts (muscle tissue) is about 2 (EU 2008). However, even if a factor of 2 is applied to translate filet concentrations to whole fish, the majority of the data met the EQS. The only exceptions were again bream from the Tees River (2007–2012) and fish from Mersey River in the years 2007 and 2008.

Comparability and applicability of the monitoring data can be enhanced by normalizing the data, e.g., to 5% lipid in the case of lipophilic compounds as proposed by the EU guidance document for the implementation of the WFD biota monitoring (EC 2014). The fish analyzed here had lipid levels of ≤5% (Table S3, Electronic Supplementary Material) so normalization results in higher HBCD levels. However, with the exception of bream from the rivers Tees (all years; range 177–725 μg kg^−1^) and Mersey (2008, 181 μg kg^−1^), all fish met the WFD EQS even after 5% lipid normalization.

Taken together, the available monitoring data indicate that the EU WFD EQS (to be implemented by end of 2018) will only be exceeded at a few sites in Europe (i.e., near point sources or at legacy sites). Since the EU EQS is an overall quality standard, it considers not only secondary poisoning but also other protection goals. The quality standard for the protection of the pelagic community (0.31 μg L^−1^; EU 2011), for example, is by a factor of about 190 higher than the water concentration that corresponds to the HBCD EQS fish tissue concentration of 167 μg kg^−1^ (= 0.0016 μg L^−1^; EU 2011).

### HBCD diastereomer patterns and concentrations in SPM/sediment

SPM samples collected in the current study are annual composite samples from four 3-month sampling periods. Table S6 (Electronic Supplementary Material) summarizes the sample characteristics.

**Fig. 2:**
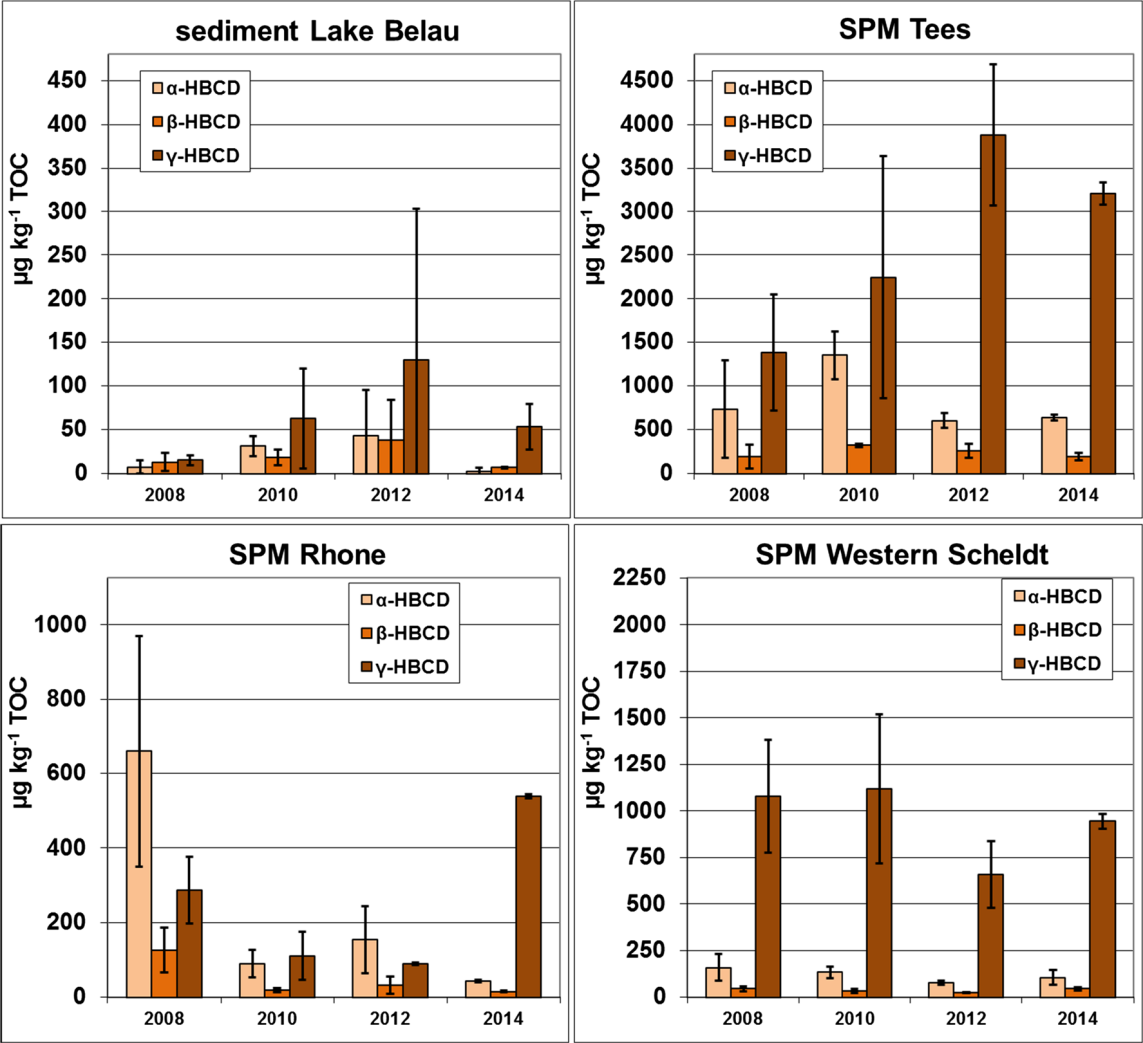
Concentrations of α-, β-, and γ-HBCD in sediment from Lake Belau and SPM from sites at the rivers Tees, Rhône, and Western Scheldt (organic carbon-normalized data; μg kg^−1^ TOC). The standard deviations are derived from replicate analyses of the same annual pool sample (*n* = 2–6; measure of analytical reproducibility) or four 3-month samples for Tees 2008 and Rhône 2010 (measure of seasonal variability)

A recent study supported the principal applicability of (continuously sampled) SPM for trend monitoring (Schubert et al. 2012). Direct comparison of SPM and sediment concentrations may, however, be limited because of differences in particle size fractions. This can be overcome by normalization to a certain particle size fraction (Schubert et al. 2012) or—for non-polar organic compounds—normalization to the total organic carbon (TOC) content (Law et al. 2008, di Toro et al. 1991). HBCD has a high log *K*_OW_ and is expected to adsorb to the organic carbon fraction. Thus, HBCD concentrations of sediment and SPM were normalized to the TOC content to enhance comparability between sites and sampling times (Fig. 2; Tables S7 and S8, Electronic Supplementary Material).

SPM/sediment was analyzed for the three main HBCD diastereomers (degradation products were not covered). Averaged over all sites and years, 23% α-HBCD (range 6–58%), 10% β-HBCD (range 3–32%), and 67% γ-HBCD (range 31–90%) were detected (Table 2). The fact that the dominant diastereomer in most SPM and sediment samples was γ-HBCD is in line with, e.g., Covaci et al. (2006), Hloušková et al. (2014), and Harrad et al. (2009).

**Table 2:** HBCD diastereomer pattern for suspended particulate matter (rivers) and sediment (Lake Belau). Fractions of HBCD diastereomers in %

Site	Mean (range) of diastereomer fraction			Remark
	α-HBCD	β-HBCD	γ-HBCD	
Lake Belau sediment	20% (6–32%)	20% (11–32%)	60% (49–82%)	Highest fraction of γ-HBCD in 2014
Götaälv SPM	56%	11%	33%	Sampling only in 2008
Tees SPM	24% (13–37%)	7% (5–9%)	69% (54–81%)	
Rhône SPM	39% (7–58%)	8% (3–11%)	52% (31–90%)	Highest fraction of γ-HBCD in 2014
Mersey SPM	11%	3%	86%	Sampling only in 2008
Western Scheldt SPM	11% (9–12%)	3% (3–4%)	85% (84–86%)	–

There were, however, individual SPM samples from the Rhône and the Götaälv that contained relatively high fractions of α-HBCD (Table 2, note that SPM at the Götaälv was only sampled in 1 year). At the Rhône site, the diastereomer pattern varied between years and γ-HBCD dominated only in 2014. SPM from the Western Scheldt contained about 85% γ-HBCD in all years. In SPM samples from the Tees, the α-HBCD fraction decreased from about 30–40% in the years 2008 and 2010 to 13% in 2012 and remained roughly at this level (in 2014: 16%). Lake Belau sediment contained mainly γ-HBCD (increase from 49% in 2008 to 59% in 2012 and 82% in 2014) while α- and ß-HBCD fractions were in the range of about 10 to 30%. The observed changes of ΣHBCD levels in the lake sediment (higher levels 2010 and 2012) did not alter the pattern.

Concentrations of ΣHBCD in SPM/sediment were the highest in the Mersey (only data for 2008) and the Tees and the lowest in Lake Belau (Fig. 2 and Table S8, Electronic Supplementary Material). Strong seasonal variations were observed in the Rhône in 2012 and the Tees in 2008 (Table S7, Electronic Supplementary Material).

In the Rhône, ΣHBCD levels in SPM decreased by about 75% between 2008 and 2010/2012 (1070 vs. 277 μg kg^−1^ TOC in 2008 and 2012, respectively; Table S8, Electronic Supplementary Material). In 2014, however, an increase to 598 μg kg^−1^ TOC was detected that went hand in hand with a change in diastereomer pattern: while α-HBCD accounted for about 40–60% in 2008–2012, γ-HBCD dominated in 2014 contributing 90% to ΣHBCD.

Temporal changes were similar at the Western Scheldt with decreasing HBCD levels in SPM between 2008 and 2012 (ΣHBCD: decrease from 1280 to 761 μg kg^−1^ TOC) followed by an increase to 1100 μg kg^−1^ TOC in 2014. The 2008 sample from the Scheldt was used as laboratory reference material and analyzed nine times during the project period. Among these, three outliers identified by the Grubbs test were eliminated (see Table S7, Electronic Supplementary Material).

In contrast, HBCD levels in SPM from the Tees increased between 2008 and 2012 (2320 vs. 4750 μg kg^−1^ TOC). In November 2012, a massive flood occurred in the Tees which may have influenced the last sampling in that year (e.g., by remobilization of HBCD-contaminated sediment from upstream regions). By 2014, levels had decreased again to 4040 μg kg^−1^ TOC.

In addition to SPM, sediment was sampled in the Tees in 2013 (Table S9, Electronic Supplementary Material). The respective ΣHBCD levels were comparable to those of SPM sampled in 2014. There was no significant difference in ΣHBCD levels between sediment taken upstream and downstream of the barrage, the latter being an area influenced by inflowing North Sea water. However, the upstream sample displayed higher α- and β-HBCD fractions (13 and 9% vs. 6 and 3%, respectively).

Sediment from Lake Belau was generally less contaminated. Nevertheless, an increase in ΣHBCD was observed from 36 μg kg^−1^ TOC in 2008 to 212 μg kg^−1^ TOC in 2012. In 2014, HBCD levels had decreased again to 63 μg kg^−1^ TOC. HBCD is obviously not evenly distributed in the lake sediment leading to high standard deviations 2010 and 2012 (Table S8, Electronic Supplementary Material).

The variability between replicate analyses of SPM samples was always relatively high. The reason for this heterogeneity is currently unknown. The freeze-dried and pooled sample material was a homogeneous powder of small particles. Routinely relatively large amounts of about 3 g SPM were applied for the SPM analysis which usually compensates sufficiently for inhomogeneity. Experience from SPM samples investigated in the German ESB program treated in the same manner showed that the homogenization procedure is adequate for, e.g., analysis of metals and chlorinated legacy compounds. Possibly, a few particles with high HBCD loads are causing the high levels found in some samples (for example, remobilized sediment from sites with higher concentrations or microplastic particles containing HBCD; see e.g., Haukås et al. 2010, Jang et al. 2016).

### Temporal changes of HBCD levels in SPM/sediment

Figure S2 (Electronic Supplementary Material) presents the temporal comparison of HBCD concentrations and patterns in SPM/sediment at four sites. Diastereomer patterns differed between sites but were mostly quite consistent for one site.

The non-parametric Mann-Kendall test did not detect any significant trends in SPM or sediment for ΣHBCD concentrations or the levels of the individual HBCD diastereomers. Due to the short time series, the LOESS approach could not be applied here.

### EQS compliance of SPM

For HBCD, the compliance of waters under the EU WFD is assessed on basis of the biota EQS (EU 2013, EU 2011). While for other priority substances a conversion of the EQS from one matrix to another is possible by applying the equilibrium partitioning approach (di Toro et al. 1991), this seems not appropriate for HBCD since organisms like fish are capable of bio-transforming HBCD diastereomers taken up from water or sediment (which eventually leads to high levels of α-HBCD in biota). However, in the EQS dossier for HBCD (EU 2011), a sediment EQS of 860 μg kg^−1^ dw was derived (for sediments with 5% TOC; corresponding to 17,200 μg kg^−1^ TOC for ΣHBCD) based on a ecotoxicity test with *Lumbriculus variegatus* (28-day no-observed effect concentration of 8.6 mg kg^−1^ dw; endpoint: total number of worms; assessment factor of 10). This TOC-normalized EQS can be applied to SPM assuming that SPM is comparable to freshly deposited sediment.

The HBCD sediment EQS of 17,200 μg kg^−1^ TOC is not exceeded at any of the investigated sites. However, the ΣHBCD levels in SPM from the Mersey were only about 5% below this threshold.

### Comparison of HBCD levels in SPM/sediment with data reported in the literature

To date, no published data for HBCD in SPM are available. However, SPM can be regarded as similar to the most recently formed sediment (on the one hand, SPM can be the result of re-suspension of the upper sediment layer at upstream sites; on the other hand, it is anticipated to be the future upper sediment layer at sites downstream). For comparison with published data, it is therefore assumed that SPM levels of HBCD correspond to surface sediment concentrations. The comparison focuses on monitoring data from Europe and refers to dry weight data where available.

For Lake Belau sediment, a direct comparison of monitoring data is possible. Stiehl et al. (2008) reported ∑HBCD concentrations of 1.9 μg kg^−1^ dw in sediment from Lake Belau sampled in 2002/2003. This is about a factor of 2 lower than the levels detected in the present study in 2008 (i.e., 3.7 ± 2.5 μg kg^−1^ dw). Relatively low sediment concentrations are also reported for English lakes with 0.88–4.8 μg kg^**−1**^ dw ∑HBCD (with 60–80% γ-HBCD, Harrad et al. 2009) and for Lake Thun (Switzerland) with total HBCD levels of up to about 60 μg kg^−1^ TOC (Bogdal et al. 2008). The latter level is in the lower range of TOC-based HBCD concentrations for Lake Belau sediment (36–212 μg kg^−1^ TOC for the period 2008–2014).

Higher HBCD levels are reported for river sediments in industrialized regions, e.g., the Scheldt near Antwerp (up to 950 μg kg^−1^ dw; Morris et al. 2004) or the rivers Skerne and Tees (up to 1680 and 511 μg kg^−1^ dw, respectively; Morris et al. 2004). Along the course of rivers, sediment levels of HBCD seem to increase downstream of industrialized sites, e.g., in River Cinca, Spain (highest value 514 μg kg^−1^ dw; Eljarrat et al. 2004). Guerra et al. (2008) reported HBCD sediment concentrations in the Cinca River in the range of <2–2660 μg kg^−1^ with γ-HBCD as major diastereomer (up to 90%). In the current study, comparably high levels were only detected in SPM from the Mersey River (about 1300 μg kg^−1^ dw ΣHBCD in 2008).

Barber et al. (2014) analyzed HBCD diastereomers in sediments sampled in the UK between 2010 and 2012. They detected HBCD only at sites in the northeast of England. Concentrations were mostly <1 μg kg^−1^ dw ΣHBCD with γ-HBCD as dominant diastereomer. In sediment from the mouth of the Tees River, HBCD concentrations were around 15 μg kg^−1^ dw and thus much lower than those found in Tees sediments and SPM in the present study (ΣHBCD levels of several hundred μg kg^−1^ dw). In sediment from Mersey River, Barber et al. (2014) detected γ-HBCD at levels just above the respective LOQ (<0.1–0.5 μg kg^−1^ dw) which also is clearly lower than the SPM levels in the present study (about 1300 μg kg^−1^ dw ΣHBCD). However, since no exact sampling locations were reported, it could not be clarified whether different sites could explain the difference. Hloušková et al. (2014) investigated sediments sampled at different locations of the Czech Republic in 2010. HBCD was found in 30 of 31 samples with γ-HBCD as the predominant diastereomer in most samples. High ∑HBCD concentrations of 30–40 μg kg^−1^ dw were detected, e.g., in the Lužická Nisa River (similar to ∑HBCD levels in SPM sampled at the Western Scheldt in the present study) while lowest levels were <1 μg kg^−1^ dw. Luigi et al. (2015) investigated sediment samples taken at five sites in the Po River in Italy. The highest levels around 10.4 μg kg^−1^ dw ∑HBCD were detected at the confluence with the Lambro River (comparable to the Rhône data in the present study in recent years). Concentrations decreased downstream to 0.84 μg kg^−1^ dw while the lowest levels were detected in sediment from a site upstream of the confluence (0.22 μg kg^−1^ dw). The main stereoisomer was γ-HBCD while α-HBCD contributed up to 25% of the total HBCD concentration.

The data suggest that the HBCD diastereomer pattern and the ∑HBCD levels strongly depend on site characteristics (e.g., specific emission situations). In general, the HBCD levels detected in sediment and SPM samples in the present investigation fit well in the range of HBCD sediment concentrations reported in literature.

### Synopsis of fish and SPM/sediment data

For the present study, river sampling sites near the mouths or in estuaries were selected. It is assumed that these sites are representative of emissions from HBCD point sources (e.g., textile industry in the Scheldt area) as well as of diffuse inputs entering the rivers by, e.g., WWTP effluents (Rüdel et al. 2012).

Bream (*A. brama*) was chosen as monitoring organism because of its mostly sedentary behavior and wide-spread presence in European rivers (Froese and Pauly 2016; Rüdel et al. 2012). It is considered as an appropriate candidate for biota monitoring especially when spatial comparisons are intended. Bream are bottom feeders and thus potentially exposed to contaminants like HBCD for which the sediment is the main sink in waters. Bream do not feed on SPM, but SPM is connected to the sediment via deposition/re-suspension of the surface sediment layer (although this may be different in areas upstream of barrages as is the case in the Tees River where the water depth is up to 4 m).

The current investigation and previous studies indicate that the HBCD diastereomer distributions in fish and SPM/sediment are different. The α-diastereomer typically dominates in fish, whereas γ-HBCD is usually the major diastereomer in sediment and SPM.

The fish and sediment/SPM monitoring data principally allow the estimation of biota-sediment accumulation factors or biota-suspended solids accumulation factors (BSAFs/BSSAFs; Burkhard et al. 2012). However, in the case of HBCD, it has to be considered that fish are capable of diastereomer-specific bio-transformation (isomerization/degradation). Moreover, the study of Yang et al. (2016) suggests that HBCD is also degraded in the sediment: the authors showed that the already relatively high fraction of α-HBCD in sediment core samples from English lakes increased further with depth. This was attributed to within-sediment, post-depositional isomerization as well as to different degradation rates among the HBCD diastereomers (Yang et al. 2016). Thus, an estimation of BSAFs/BSSAFs seems only meaningful for ΣHBCD but not for the single diastereomers.

Calculations were possible for the years where samplings for both matrices overlapped (2008, 2010, 2012). Detailed data are listed Table S10 (Electronic Supplementary Material). BSAFs/BSSAFs differed between sites as well as between years at most sites. At most sites, BSAFs/BSSAFs were in the range of 0.1–4. Data from Lake Belau are noticeable because levels in sediment and fish deviated between years and BSAF values reversed: while in 2008, levels were clearly higher in bream, it was the other way around in 2010 and 2012 (Fig. S2, Electronic Supplementary Material). Accordingly, the BSAF of ΣHBCD in Lake Belau fish decreased from about 16 in 2008 to 0.1 in 2012. For the Tees, increasing levels of ΣHBCD in SPM but only slight variations in bream resulted in decreasing BSSAFs, from 4.1 in 2008 to 2.1 in 2012. BSSAFs in the Rhône were constantly about 1 mirroring the simultaneous decrease of ΣHBCD in fish and SPM at this site. In the rivers Götaälv and Mersey, the ΣHBCD load of SPM exceeded that of bream by far leading to low BSSAFs of 0.3 and 0.2, respectively, for 2008 (only in this year, SPM was sampled). The lowest BSSAFs, however, were estimated for bream (range 0.03–0.06) and sole (0.01–0.14) at the Western Scheldt. This may be indicative of a lower availability or uptake of HBCD under brackish water conditions. Figure S2 (Electronic Supplementary Material) integrates the temporal comparison of HBCD levels and patterns in fish and SPM/sediment for those sites that have been sampled continuously.

In summary, the data suggest that the bioaccumulation potential of HBCD via sediment/SPM-bound HBCD is relatively low at freshwater sites. In previous studies on HBCD, freshwater BSAFs in the same range were reported, i.e., 0.10–1.44 for barbel and bleak from Spanish rivers (van Beusekom et al. 2006), and 0.60 and 15 for pike from two sites at the Swedish River Viskan (Sellström et al. 1998).

The question of whether SPM or a fish-based monitoring is most appropriate to detect environmental trends of HBCD diastereomers is difficult to answer. Due to their adsorptive and lipophilic properties, HBCD diastereomers distribute into several environmental compartments. In the case of bream and SPM, there seems to be no direct trophic link. Consequently, a trend in bream is probably not indicative for the SPM and vice versa. Moreover, HBCD diastereomer levels in both compartments are likely to be influenced by different factors (e.g., abiotic degradation, adsorption, isomerization, metabolism, excretion). Taken together, bream seem to be a suitable monitor for the bioavailability of HBCD while SPM might be more appropriate for the following of inputs of (particle-bound) HBCD.

## Conclusions

The fish and sediment/SPM data indicate that in recent years, environmental HBCD burdens are declining at those sites that are subject to diffuse emissions. This was to be expected because of the emission control measures implemented by HBCD producers and users in Europe (VECAP 2015). While at some sites (Rhône, Western Scheldt), a concomitant decrease in fish and SPM was detectable, the data were less conclusive at others (Lake Belau, Tees).

The current study confirms the preliminary fish trend data based on the first 4 years reported in Rüdel et al. (2012). The decrease of ΣHBCD fish concentrations, however, slowed down in recent years as compared to the changes observed in the period 2007–2010.

This study allows a first comparative view on the compliance with the HBCD biota EQS given in WFD daughter directive 2013/39/EU (EU 2013) in rivers from several European countries. It shows that, with the exception of one contaminated site, fish complied with the biota EQS for HBCD.

## Electronic supplementary material

ESM 1 (DOCX 586 kb)
