# Supplementary material for: Hexabromocyclododecane diastereomers in fish and suspended particulate matter from selected European waters—trend monitoring and environmental quality standard compliance
Source: Environ Sci Pollut Res Int. 2017 Jun 17;24(22):18048–62. doi: 10.1007/s11356-017-9469-4 (PMC5554270; doi:10.1007/s11356-017-9469-4)
Supplement: Supplementary file 1 — (DOCX 586 kb) [file 11356_2017_9469_MOESM1_ESM.docx]

**Electronic Supplementary Material**

**Hexabromocyclododecane diastereomers in fish and suspended particulate matter from selected European waters - trend monitoring and environmental quality standard compliance**

Heinz Rüdel^1^*, Josef Müller^2^, Jens Nowak^2^, Mathias Ricking^3^, Roland Klein^4^, Matthias Kotthoff^2^

1 Fraunhofer Institute for Molecular Biology and Applied Ecology (Fraunhofer IME), Department Environmental Specimen Bank and Elemental Analysis, 57392 Schmallenberg, Germany

2 Fraunhofer Institute for Molecular Biology and Applied Ecology (Fraunhofer IME), Department Environmental and Food Analysis, 57392 Schmallenberg, Germany

3 Department Earth Sciences, Anthropocene Research, Geochemistry, Freie Universität Berlin, 12249 Berlin, Germany

4 Biogeography, University of Trier, 54286 Trier, Germany

* Corresponding author: e-mail: [heinz.ruedel@ime.fraunhofer.de](mailto:heinz.ruedel@ime.fraunhofer.de); phone: +49 2972 302 301

**Fish Yield and Biometric Data**

Table S1 (Electronic Supplementary Material, see below) lists the number of fish per annual sampling and the respective biometric data (i.e., mean and standard deviation of age, weight, length, and condition factor, and sex ratios of annual fish pools). The desired number of 15 fish was achieved at nearly all samplings. An exception was the Mersey River (UK) where mostly less and smaller fish were caught. At this site bream were re-introduced only recently so that populations were still small with mainly young individuals. In 2007 and 2009 fish numbers were also low at the Western Scheldt. From 2009 onwards the bream sampling at Western Scheldt had been shifted from September to late autumn when gillnet fishing is allowed in order to increase catches. Due to the partly low abundance of bream in the Western Scheldt a second fish species was included in the sampling scheme. At this site also sole (*Solea solea*) was sampled, a species well adapted to brackish estuarine waters.

The sampling at the Tees River (UK) in 2013 yielded bream with a significantly lower weight and size. This is probably related to a major flood event in that region in the previous year (winter 2012/2013) which removed most of the sediment fraction < 2 mm from the sampling area (see discussion in the main paper, section `HBCD Diastereomer Patterns and Concentrations in Fish’). Since bream feed on sediment, this probably reduced the food availability causing decreased fish abundances as well as reduced fish sizes due to migration (see discussion in the main paper, section `HBCD Diastereomer Patterns and Concentrations in Fish’). On the other hand, fish health parameters such as fat content and condition factor (see below) of the bream from 2013 were comparable to those of the previous years.

The sex ratio (no. females / no. males) at the selected sites varied from year to year. Mostly more female bream were caught. Especially at the Tees, Western Scheldt and Lake Belau sites female bream dominated and sex ratios were high in some years (up to 14 at Tees and up to 6.5 at Lake Belau). In the samples from the Mersey and Rhône sex ratios varied between 0.3 - 2.8. A consistently higher number of males (i.e., a sex ratio < 1) was only detected at the Götaälv.

Condition factors for bream were between 1.0 and 1.4 g cm^-3^ (Table S1). Variations between years were relatively low indicating that fish health did not change significantly in the investigated period.

The mean condition factors for Western Scheldt sole were mostly in the range 0.92 - 0.98 g cm^-3^. A clearly lower condition factor of 0.80 g cm^-3^ was only detected in sole sampled in 2011. For juvenile sole condition factors of 1.1 - 1.2 g cm^-3^ are reported by Amara et al. (2007) whereas factors below 0.9 g cm^-3^ are considered as insufficient reflecting metabolic alterations caused by food shortage. Assuming that condition factors of juvenile sole and just mature sole as sampled here are comparable, sole caught in 2011 seemed to be in a relatively bad condition. On the other hand, sole from this year had the highest fat content of all years which may be interpreted as an indicator of a good nutrition situation. In spite of these differences in biometric factors, the HBCD concentrations for sole from 2011 were not strikingly different in comparison to the other years and thus the data were used for the further evaluations.

**References cited in this Electronic Supplementary Material**

Amara R, Meziane T, Gilliers C, Hermel G, Laffargue P (2007): Growth and condition 350 indices in juvenile sole Solea solea measured to assess the quality of essential fish 351 habitat. Mar Ecol Prog Ser 351: 201-208.

Nguetseng R, Fliedner A, Knopf B, Lebreton B, Quack M, Rüdel H (2015): Retrospective monitoring of mercury in fish from selected European freshwater and estuary sites. Chemosphere 134:427-434.

Rüdel H, Müller J, Quack M, Klein R (2012): Monitoring of hexabromocyclododecane diastereomers in fish from European freshwaters and estuaries. Environ Sci Pollut Res 19:772-783.

Table S1: Number of individuals per sampling and biometric data of fish (sex ratio, age, weight, length, condition factor + standard deviations). In the first column beneath each site name the geographical coordinates are given. Data are from Rüdel et al. (2012) and Nguetseng et al. (2015).

| **river/lake, geocoor­dinates** | **year** | **no. of fish** | **sex ratio  (no. females / no. males)** | **mean age + std. dev. [years]** | **mean weight + std. dev. [g]** | **mean length + std. dev. [cm]** | **mean condition factor + std. dev.  [g cm^-3^] ¥** |
| --- | --- | --- | --- | --- | --- | --- | --- |
| Tees, UK | 2007 | 15 | nd**#** | 8 + 2 | 1320 + 460 | 47 + 6 | 1.2 + 0.1 |
| 54°33′ N, | 2008 | 15 | 0.7 | 9 + 1 | 1330 + 300 | 48 + 3 | 1.2 + 0.1 |
| 1°18′ W | 2009 | 15 | 14 | 9 + 2 | 1300 + 320 | 48 + 4 | 1.1 + 0.1 |
|  | 2010 | 15 | 1.5 | 8 + 3 | 1100 + 580 | 45 + 7 | 1.1 + 0.1 |
|  | 2011 | 15 | 4.0 | 11 + 1 | 1350 + 300 | 50 + 4 | 1.0 + 0.1 |
|  | 2012 | 14 | 0.8 | 10 + 2 | 1280 + 360 | 48 + 5 | 1.2 + 0.1 |
|  | 2013 | 14 | 3.7 | 8 + 4 | 704 + 482 | 37 + 7 | 1.2 + 0.1 |
| Mersey, | 2007 | 11 | nd | 3 + 1 | 329 + 62 | 31 + 2 | 1.1 + < 0.1 |
| UK § | 2008 | 15 | 1.5 | 5 + 1 | 509 + 92 | 35 + 2 | 1.1 + 0.1 |
| 53°23′ N, | 2012 | 11 | 1.2 | 4 + 1 | 448 + 166 | 33 + 4 | 1.2 + < 0.1 |
| 2°34′ W | 2013 | 10 | 0.3 | 7 + 2 | 755 + 324 | 38 + 5 | 1.3 + 0.1 |
| Götaälv, | 2007 | 15 | nd | 9 + 3 | 1210 + 360 | 47 + 5 | 1.2 + 0.1 |
| SE § | 2008 | 15 | 0.7 | 10 + 4 | 1280 + 380 | 46 + 4 | 1.3 + 0.1 |
| 57°43′ N, | 2012 | 15 | 0.5 | 12 + 2 | 1660 + 200 | 52 + 2 | 1.2 + 0.1 |
| 11°59′ E | 2013 | 15 | 0.9 | 12 + 2 | 1590 + 280 | 48 + 3 | 1.4 + 0.1 |
| Rhône, | 2007 | 15 | nd | 4 + 1 | 405 + 108 | 32 + 3 | 1.2 + 0.1 |
| FR | 2008 | 15 | 1.5 | 7 + 1 | 760 + 200 | 41 + 4 | 1.1 + 0.1 |
| 43°38′ N, | 2009 | 15 | 1.5 | 6 + 1 | 646 + 131 | 37 + 3 | 1.3 + 0.1 |
| 4°36′ E | 2010 | 15 | 0.7 | 5 + 1 | 609 + 230 | 37 + 4 | 1.2 + 0.1 |
|  | 2011 | 15 | 0.9 | 7 + 1 | 810 + 263 | 41 + 4 | 1.1 + 0.2 |
|  | 2012 | 15 | 2.8 | 7 + 1 | 814 + 198 | 42 + 4 | 1.1 + 0.1 |
|  | 2013 | 15 | 1.1 | 6 + 1 | 589 + 82 | 36 + 2 | 1.2 + 0.2 |
| Lake | 2007 | 15 | nd | 9 + 4 | 1010 + 380 | 44 + 6 | 1.1 + 0.1 |
| Belau, | 2008 | 15 | 6.5 | 10 + 4 | 1480 + 380 | 50 + 4 | 1.2 + 0.1 |
| DE | 2009 | 15 | 6.5 | 13 + 2 | 1950 + 570 | 54 + 5 | 1.2 + 0.1 |
| 54°06′ N, | 2010 | 15 | 0.9 | 10 + 4 | 1330 + 520 | 48 + 7 | 1.1 + 0.1 |
| 10°15′ E | 2011 | 15 | 6.5 | 12 + 3 | 1630 + 310 | 54 + 4 | 1.0 + 0.2 |
|  | 2012 | 15 | 2.0 | 13 + 3 | 1690 + 240 | 55 + 3 | 1.0 + 0.1 |
|  | 2013 | 15 | 2.8 | 13 + 3 | 1840 + 260 | 55 + 3 | 1.1 + 0.1 |
| Western | 2007-9 | 7 | 2.5 | 9 + 3 | 1470 + 480 | 49 + 6 | 1.2 + 0.1 |
| Scheldt, | 2008-9 | 15 | 1.1 | 9 + 4 | 1480 + 410 | 50 + 7 | 1.2 + 0.2 |
| NL§, | 2009-11 | 15 | 2.8 | 8 + 2 | 1420 + 220 | 50 + 3 | 1.1 + 0.1 |
| bream | 2010-11 | 15 | 1.5 | 9 + 2 | 1490 + 240 | 51 + 4 | 1.1 + 0.1 |
| 51°23′ N, | 2011-11 | 15 | 2.0 | 10 + 3 | 1720 + 340 | 53 + 4 | 1.1 + 0.1 |
| 4°14′ E | 2012-11 | 15 | 4.0 | 12 + 3 | 2230 + 300 | 58 + 3 | 1.1 + 0.1 |
|  | 2013-11 | 9 | 2.0 | 11 + 3 | 1930 + 480 | 53 + 4 | 1.3 + 0.2 |
| Western, | 2007 | 15 | nd | approx. 3 | 239 + 24 | 29 + 1 | 0.98 + 0.09 |
| Scheldt, | 2008 | 15 | nd | > 3 | 396 + 82 | 34 + 2 | 0.96 + 0.09 |
| NL | 2009 | 15 | nd | > 3 | 438 + 169 | 35 + 4 | 0.98 + 0.07 |
| **sole** | 2010 | 15 | nd | > 3 | 300 + 116 | 31 + 4 | 0.97 + 0.07 |
| 51°23′ N, | 2011 | 15 | nd | > 3 | 177 + 55 | 28 + 2 | 0.80 + 0.08 |
| 4°14′ E | 2012 | 15 | nd | > 3 | 298 + 57 | 32 + 2 | 0.92 + 0.07 |

# sex of bream was not determined at the first sampling in 2007; no sex determination possible for sole. § from 2009 onwards sampling was shifted to late autumn since in this period gillnet sampling is allowed. ¥ the condition factor was calculated as 100 * body weight [g] / (length [cm])^3^.

Table S2: Concentrations of α-, β- and γ-HBCD in bream muscle samples from the samp­ling period 2007 to 2013 (**wet weight data**). Data for the period 2007 - 2010 are from Rüdel et al. (2012).
stand. dev. = standard deviation. For data below LOQ (LOD) measured values are reported and used for ΣHBCD and mean value calculations. Data < LOQ are in parentheses (< LOD in italics). LOQ = 0.1 µg kg^-1^ ww, LOD = 0.03 µg kg^-1^ ww.

| **year** | **sample** |  |  | **HBCD [µg kg**^-1^**]  wet weight** |  | **ΣHBCD** |
| --- | --- | --- | --- | --- | --- | --- |
|  |  |  | **α-HBCD** | **β-HBCD** | **γ-HBCD** | **[µg kg**^-1^**]** |
| **bream samples Lake Belau (DE), 2007 - 2013** | | | | | | |
| 2007 | Lake Belau (07/B/D/B/001) | mean | 1.3 | 0.7 | 4.4 | 6.4 |
|  | (n = 4) | stand. dev. | 1.0 | 0.5 | 3.2 | 4.7 |
| 2008 | Lake Belau (08/B/D/B/001) | mean | 1.8 | 1.2 | 6.5 | 9.5 |
|  | (n =15, individual fish) | stand. dev. | 4.5 | 3.0 | 15.2 | 22.6 |
| 2009 | Lake Belau (09/B/D/B/002+007) | mean | 0.2 | *(0.03)* | (0.08) | 0.3 |
|  | (n = 3) | stand. dev. | 0.02 | 0.02 | 0.04 | 0.1 |
| 2010 | Lake Belau (10/B/D/B/002) | mean | 0.4 | 0.2 | 0.8 | 1.4 |
|  | (n = 2) | stand. dev. | 0.1 | 0.1 | 0.6 | 0.7 |
| 2011 | Lake Belau (11/B/D/B/014) | mean | 0.1 | (0.04) | 0.3 | 0.4 |
|  | (n = 2) | stand. dev. | 0.04 | 0.01 | 0.2 | 0.2 |
| 2012 | Lake Belau (12/B/D/B/003) | mean | (0.09) | *(0.03)* | 0.2 | 0.3 |
|  | (n = 2) | stand. dev. | 0.03 | 0.01 | 0.1 | 0.2 |
| 2013 | Lake Belau (13/B/D/B/001) | mean | 0.9 | 0.5 | 2.7 | 4.1 |
|  | (n = 2) | stand. dev. | 0.1 | 0.03 | 0.1 | 0.04 |
| **bream samples Götaälv (SE), 2007, 2008, 2012, 2013** | | | | | | |
| 2007 | Götaälv (07/B/S/G/001 ) | mean | 0.9 | (0.03) | 0.2 | 1.1 |
|  | (n = 2) | stand. dev. | 0.1 | 0.01 | 0.0 | 0.1 |
| 2008 | Götaälv (08/B/S/G/001+007) | mean | 1.9 | 0.2 | 0.8 | 2.9 |
|  | (n = 3) | stand. dev. | 0.3 | 0.1 | 0.7 | 1.2 |
| 2012 | Götaälv (12/B/S/G/003 ) | mean | 3.1 | 0.9 | 5.1 | 9.1 |
|  | (n = 2) | stand. dev. | 0.1 | 0.1 | 0.7 | 0.8 |
| 2013 | Götaälv (13/B/S/G/001) |  | 1.9 | *(0.02)* | (0.08) | 2.0 |
|  | (n = 1) |  |  |  |  |  |
| **bream samples Tees (UK), 2007 - 2013** | | | | | | |
| 2007 | Tees (07/B/UK/T/001) | mean | 358 | 4.7 | 11.1 | 374 |
|  | (n = 2) | stand. dev. | 16 | 0.2 | 2.1 | 18 |
| 2008 | Tees (08/B/GB/T/001) |  | 310 | 3.7 | 5.6 | 320 |
|  | (n = 1) |  |  |  |  |  |
| 2009 | Tees (09/B/GB/T/007) |  | 377 | 3.4 | 5.5 | 386 |
|  | (n = 1) |  |  |  |  |  |
| 2010 | Tees (10/B/UK/T/002) |  | 212 | 1.2 | 2.0 | 215 |
|  | (n = 1) |  |  |  |  |  |
| 2011 | Tees (11/B/GB/T/014) | mean | 239 | 2.9 | 4.2 | 246 |
|  | (n = 2) | stand. dev. | 6 | 0.8 | 0.2 | 7 |
| 2012 | Tees (12/B/GB/T/003) | mean | 239 | 2.7 | 6.1 | 247 |
|  | (n = 2) | stand. dev. | 2 | 0.4 | 2.2 | 1 |
| 2013 | Tees (13/B/GB/T/001) | mean | 71.2 | 1.3 | 2.5 | 75.0 |
|  | (n = 2) | stand. dev. | 0.5 | 0.2 | 0.5 | 0.2 |
| **bream samples Rhône (FR), 2007 - 2013** | | | | | | |
| 2007 | Rhône (07/B/F/R/001) | mean | 9.6 | (0.1) | 0.9 | 10.6 |
|  | (n = 4) | stand. dev. | 0.8 | 0.05 | 0.3 | 0.8 |
| 2008 | Rhône (08/B/F/R/001) |  | 27.2 | 0.3 | 2.7 | 30.2 |
|  | (n = 1) |  |  |  |  |  |
| 2009 | Rhône (09/B/F/R/007) |  | 16.9 | 0.2 | 2.3 | 19.3 |
|  | (n = 1) |  |  |  |  |  |
| 2010 | Rhône (10/B/F/R/002) | mean | 4.4 | (0.1) | 0.5 | 5.0 |
|  | (n = 2) | stand. dev. | 0.2 | 0.02 | 0.002 | 0.2 |
| 2011 | Rhône (11/B/F/R/014) | mean | 2.4 | 0.2 | 1.5 | 4.1 |
|  | (n = 2) | stand. dev. | 0.4 | 0.2 | 1.4 | 2.0 |
| 2012 | Rhône (12/B/F/R/003) | mean | 6.5 | 0.3 | 0.6 | 7.4 |
|  | (n = 2) | stand. dev. | 2.2 | 0.4 | 0.2 | 2.8 |
| 2013 | Rhône (13/B/F/R/001) | mean | 3.0 | (0.09) | 0.7 | 3.8 |
|  | (n = 2) | stand. dev. | 0.2 | 0.04 | 0.3 | 0.6 |
| **bream samples Mersey (UK), 2007, 2008, 2012, 2013** | | | | | | |
| 2007 | Mersey I (07/B/UK/M/001-I) | mean | 79.7 | 4.2 | 8.3 | 92.1 |
|  | (n = 2) | stand. dev. | 5.8 | 0.2 | 0.3 | 5.7 |
| 2008 | Mersey (08/B/UK/M/001+003) | mean | 77.7 | 4.3 | 9.3 | 91.4 |
|  | (n = 3) | stand. dev. | 4.5 | 0.1 | 0.8 | 5.5 |
| 2012 | Mersey (12/B/GB/M/003) | mean | 29.5 | 1.6 | 4.6 | 35.7 |
|  | (n = 3) | stand. dev. | 0.3 | 0.1 | 1.0 | 0.6 |
| 2013 | Mersey (13/B/GB/M/001) |  | 35.3 | 0.9 | 2.4 | 38.6 |
|  | (n = 1) |  |  |  |  |  |
| **bream samples Western Scheldt (NL), 2007 - 2013** | | | | | | |
| 2007 | Scheldt (07/B/NL/S/001-Filet1-7) | mean | 2.2 | 0.2 | 0.8 | 3.2 |
|  | (n = 2) | stand. dev. | 0.1 | 0.002 | 0.1 | 0.1 |
| 2008 | Scheldt (08/B/NL/S/001) |  | 1.8 | (0.01) | 0.2 | 1.9 |
|  | (n = 1) |  |  |  |  |  |
| 2009 | Scheldt (09/B/NL/S/006) | mean | 1.3 | (0.04) | 0.2 | 1.5 |
|  | (n = 2) | stand. dev. | 0.2 | 0.01 | 0.1 | 0.1 |
| 2010 | Scheldt (10/B/NL/S/002) |  | 0.7 | *(0.01)* | 0.1 | 0.9 |
|  | (n = 1) |  |  |  |  |  |
| 2011 | Scheldt (11/B/NL/S/014) |  | 1.1 | (0.06) | 0.3 | 1.5 |
|  | (n = 1) |  |  |  |  |  |
| 2012 | Scheldt (12/B/NL/S/003) | mean | 1.5 | (0.05) | 0.2 | 1.7 |
|  | (n = 2) | stand. dev. | 0.05 | 0.03 | 0.02 | 0.01 |
| 2013 | Scheldt (13/B/NL/S/001) |  | 1.1 | *(0.02)* | 0.2 | 1.3 |
|  | (n = 1) |  |  |  |  |  |

n - number of replicate measurements or number of individually analyzed fish (Lake Belau 2008).

Table S3: Concentrations of α-, β- and γ-HBCD in bream muscle samples from the samp­ling period 2007 to 2013 (**lipid weight data**). Data for the period 2007 - 2010 are from Rüdel et al. (2012).
stand. dev. = standard deviation. For data below LOQ (LOD) measured values are reported and used for ΣHBCD and mean value calculations. Data < LOQ are in parentheses (< LOD in italics): LOQ = 0.1 µg kg^-1^ ww or 2.0 - 13 µg kg^-1^ lw (depending on fat content),
LOD = 0.03 µg kg^-1^ ww or 0.6 - 5 µg kg^-1^ lw (depending on fat content).

| **year** | **sample** | |  | | | | |  | **HBCD** |  | **ΣHBCD** | **fat** |
| --- | --- | --- | --- | --- | --- | --- | --- | --- | --- | --- | --- | --- |
|  |  | |  | | | | | **α-HBCD** | **β-HBCD** | **γ-HBCD** |  |  |
|  |  | |  | | | | | **[µg kg**^-1^**] lipid weight** | | | | **%** |
| **bream samples Lake Belau (DE), 2007 - 2013** | | | | | | | | | | | |  |
| 2007 | Lake Belau (07/B/D/B/001) | | | mean | | | | 142 | 79 | 474 | 695 | 0.92 |
|  | n = 4 | | stand. dev. | | | | | 106 | 56 | 349 | 510 |  |
| 2008 | Lake Belau (08/B/D/B/001) | mean | | | | | | 106 | 75 | 403 | 584 | 2.2 |
|  | 15 individual fish | stand. dev. | | | | | | 271 | 184 | 938 | 1388 | 0.7 |
| 2009 | Lake Belau (09/B/D/B/00X) | | | mean | | | | 7.6 | *(0.9)* | (2.5) | 11.0 | 3.1 |
|  | n = 3 | stand. dev. | | | | | | 0.7 | 0.5 | 1.2 | 1.9 |  |
| 2010 | Lake Belau (10/B/D/B/002) | | | mean | | | | 24 | 9.8 | 50 | 84 | 1.6 |
|  | n = 2 | stand. dev. | | | | | | 6 | 4.8 | 34 | 44 |  |
| 2011 | Lake Belau (11/B/D/B/014) | | | mean | | | | 8.5 | (2.8) | 22 | 34 | 1.3 |
|  | n = 2 | stand. dev. | | | | | | 2.8 | 0.8 | 14 | 18 |  |
| 2012 | Lake Belau (12/B/D/B/003) | mean | | | | | | 8.1 | *(2.2)* | 14 | 24 | 1.1 |
|  | n = 2 | stand. dev. | | | | | | 2.7 | 1.0 | 13 | 17 |  |
| 2013 | Lake Belau (13/B/D/B/001) | mean | | | | | | 72 | 35 | 208 | 315 | 1.3 |
|  | n = 2 | stand. dev. | | | | | | 3.9 | 2.4 | 5 | 3 |  |
| **bream samples Götaälv (SE), 2007, 2008, 2012, 2013** | | | | | | | | | | | |  |
| 2007 | Götaälv (07/B/S/G/001 ) | | mean | | | | | 35 | (1.3) | 7.6 | 44 | 2.5 |
|  | n = 2 | | stand. dev. | | | | | 5 | 0.3 | 0.6 | 6 |  |
| 2008 | Götaälv (08/B/S/G/1+7) | | | | | mean | | 53 | 5.2 | 23 | 81 | 3.6 |
|  | n = 3 | stand. dev. | | | | | | 8 | 3.6 | 21 | 33 |  |
| 2012 | Götaälv (12/B/S/G/003) | | | | | mean | | 105 | 32 | 171 | 307 | 3.0 |
|  | n = 2 | stand. dev. | | | | | | 2 | 2 | 23 | 28 |  |
| 2013 | Götaälv (13/B/S/G/001) n = 1 | | | | |  | | 56 | (0.6) | (2) | 59 | 3.4 |
| **bream samples Tees (UK), 2007 - 2013** | | | | | | | | | | | | |
| 2007 | Tees (07/B/UK/T/001) | | | | mean | | | 10723 | 141 | 332 | 11196 | 3.3 |
|  | n = 2 | | | | stand. dev. | | | 465 | 6 | 63 | 535 |  |
| 2008 | Tees (08/B/GB/T/001)  n = 1 | | | |  | | | 9205 | 110 | 167 | 9482 | 3.4 |
| 2009 | Tees (09/B/GB/T/007)  n = 1 | | | |  | | | 14164 | 127 | 205 | 14496 | 2.7 |
| 2010 | Tees (10/B/GB/T/002)  n = 1 | | | |  | | | 10992 | 63 | 102 | 11157 | 1.9 |
| 2011 | Tees (11/B/GB/T/014) | | | | mean | | | 10657 | 131 | 186 | 10973 | 2.2 |
|  | n = 2 | stand. dev. | | | | | | 270 | 34 | 11 | 315 |  |
| 2012 | Tees (12/B/GB/T/003) | | mean | | | | | 9739 | 109 | 248 | 10096 | 2.4 |
|  | n = 2 | stand. dev. | | | | | | 86 | 17 | 91 | 22 |  |
| 2013 | Tees (13/B/GB/T/001) | | mean | | | | | 3359 | 60 | 117 | 3536 | 2.1 |
|  | n = 2 | | stand. dev. | | | | | 22 | 7 | 25 | 10 |  |
| **bream samples Rhône (FR), 2007 - 2013** | | | | | | | | | | | | |
| 2007 | Rhône (07/B/F/R/001) | | mean | | | | | 1285 | (10) | 122 | 1417 | 0.75 |
|  | n = 4 | | stand. dev. | | | | | 113 | 6 | 36 | 108 |  |
| 2008 | Rhône (08/B/F/R/001) n = 1 | |  | | | | | 929 | 9 | 93 | 1030 | 2.9 |
| 2009 | Rhône (09/B/F/R/007) n = 1 | |  | | | | | 463 | 6 | 62 | 531 | 3.6 |
| 2010 | Rhône (10/B/F/R/002) | | mean | | | | | 182 | (2.6) | 20.8 | 205 | 2.4 |
|  | n = 2 | | stand. dev. | | | | | 9 | 0.6 | 0.1 | 9 |  |
| 2011 | Rhône (11/B/F/R/014) | | mean | | | | | 116 | 10 | 73 | 200 | 2.1 |
|  | n = 2 | | stand. dev. | | | | | 21 | 9.7 | 68 | 99 |  |
| 2012 | Rhône (12/B/F/R/003) | | mean | | | | | 261 | 13 | 25 | 299 | 2.5 |
|  | n = 2 | | stand. dev. | | | | | 89 | 15.7 | 6 | 111 |  |
| 2013 | Rhône (13/B/F/R/001) | | mean | | | | | 174 | (5) | 43 | 222 | 1.7 |
|  | n = 2 | | stand. dev. | | | | | 10 | 2 | 20 | 32 |  |
| **bream samples Mersey (UK), 2007, 2008, 2012, 2013** | | | | | | | | | | | | |
| 2007 | Mersey I (07/B/UK/ M/001-I) | mean | | | | | | 3137 | 164 | 326 | 3627 | 2.5 |
|  | n = 2 | | stand. dev. | | | | | 229 | 6 | 10 | 224 |  |
| 2008 | Mersey (08/B/UK/ M/001+003) | | | | | | mean | 2671 | 148 | 320 | 3139 | 2.9 |
|  | n = 3 | | stand. dev. | | | | | 156 | 5 | 28 | 188 |  |
| 2012 | Mersey (12/B/GB/ M/003) | | | | | mean | | 1566 | 85 | 243 | 1894 | 1.9 |
|  | n = 2 | | stand. dev. | | | | | 14 | 5 | 53 | 33 |  |
| 2013 | Mersey (13/B/GB/ M/001)  n = 1 | | | | |  | | 1577 | 40 | 109 | 1726 | 2.2 |
| **bream samples Western Scheldt (NL), 2007 - 2013** | | | | | | | | | | | | |
| 2007 | Scheldt (07/B/NL/S/001) | | | | mean | | | 64.9 | 4.5 | 23.8 | 93.2 | 3.4 |
|  | Filets 1-7 / n = 2 | | stand. dev. | | | | | 1.6 | 0.1 | 3.2 | 1.6 |  |
| 2008 | Scheldt (08/B/NL/S/001)  n = 1 | | | |  | | | 64.3 | (0.5) | 5.6 | 70.3 | 2.7 |
| 2009 | Scheldt (09/B/NL/S/006) | | mean | | | | | 42.6 | (1.3) | 7.4 | 51.3 | 3.0 |
|  | n = 2 | | stand. dev. | | | | | 6.7 | 0.3 | 2.3 | 4.1 |  |
| 2010 | Scheldt (10/B/NL/S/002)  n = 1 | |  | | | | | 31.1 | *(0.4)* | 4.1 | 35.7 | 2.4 |
| 2011 | Scheldt (11/B/NL/S/014)  n = 1 | |  | | | | | 21.9 | (1.2) | 6.1 | 29.2 | 5.0 |
| 2012 | Scheldt (12/B/NL/S/003) | | | | | mean | | 37.7 | (1.3) | 5.8 | 44.8 | 3.9 |
|  | n = 2 | | | | stand. dev. | | | 1.2 | 0.7 | 0.6 | 0.1 |  |
| 2013 | Scheldt (13/B/NL/S/001)  n = 1 | | | | |  | | 34.6 | *(0.7)* | 4.7 | 40.0 | 3.3 |

n - number of replicate measurements or number of individually analyzed fish (Lake Belau 2008).

Table S4: Concentrations of α-, β- and γ-HBCD in sole muscle samples sampled from the Western Scheldt (NL) from 2007 to 2013. **Data are given as µg kg^-1^ wet weight**. Data for the period 2007 - 2010 are from Rüdel et al. (2012). stand. dev. - standard devia­tion.
For data below LOQ (LOD) measured values are reported and used for ΣHBCD and mean value calculations. Data below LOQ are in parentheses (data below LOD *in italics*).

|  | | | |  | **α-HBCD** | **β-HBCD** | **γ-HBCD** | **ΣHBCD** |  |
| --- | --- | --- | --- | --- | --- | --- | --- | --- | --- |
|  | | | |  | **[µg kg^-1^ wet weight]** | | | |  |
| **2007 #** | | **mean** | | | **1.6** | **0.8** | **4.7** | **7.1** |  |
| stand. dev. | | | | | 2.0 | 1.3 | 7.3 | 10.6 |  |
| **2008** | | |  | | **0.8** | **0.2** | **1.1** | **2.2** |  |
| **2009** | | |  | | **0.4** | **0.2** | **1.0** | **1.7** |  |
| **2010** | | |  | | **0.1** | **(0.02)** | **(0.06)** | **0.2** |  |
| **2011** | | |  | | **0.6** | **0.1** | **2.0** | **2.7** |  |
|  | | |  | | 0.2 | 0.13 | 0.8 | 1.1 |  |
|  | | |  | | 0.1 | (0.05) | 0.4 | 0.5 |  |
| **2012** | | | **mean** | | **0.2** | **(0.09)** | **0.6** | **0.8** |  |
|  | stand. dev. | | | | 0.1 | 0.06 | 0.3 | 0.4 |  |
|  |  | | | | *(0.00)* | *(0.01)* | *(0.00)* | *(0.01)* |  |
|  |  | | | | *(0.02)* | *(0.00)* | (0.07) | (0.10) |  |
| **2013** | **mean** | | | | ***(0.01)*** | ***(0.01)*** | **(0.04)** | **(0.06)** |  |
|  | stand. dev. | | | | *0.02* | *0.01* | 0.05 | 0.06 |  |

Table S5: Concentrations of α-, β- and γ-HBCD in sole muscle sampled from the Western Scheldt (NL) from 2007 to 2013. **Data are given as µg kg^-1^ lipid weight**. Data for the period 2007 - 2010 are from Rüdel et al. (2012). stand. dev. - standard devia­tion.
For data below LOQ (LOD) measured values are reported and used for ΣHBCD and mean value calculations. Data below LOQ are in parentheses (data below LOD *in italics*).

|  |  | **α-HBCD** | **β-HBCD** | **γ-HBCD** | **ΣHBCD** | **fat** |
| --- | --- | --- | --- | --- | --- | --- |
| **sample** |  | **[µg kg^-1^ lipid weight]** | | |  | **%** |
| sole 2007 Scheldt (07/S/NL/S/001) n = 15 | mean | 127 | 66 | 374 | 568 | 1.4 |
|  | stand. dev. | 167 | 107 | 601 | 874 | 0.6 |
| sole 2008 Scheldt (08/S/NL/S/001) n = 1 |  | 69 | 19 | 92 | 180 | 1.2 |
| sole 2009 Scheldt (09/S/NL/S/007) n = 1 |  | 53 | 26 | 123 | 202 | 0.85 |
| sole 2010 Scheldt (10/S/NL/S/002) n = 1 |  | 6.8 | (*1.4*) | (4.0) | 12 | 1.5 |
| sole 2011 Scheldt (11/S/NL/S/014) n = 1 |  | 25 | 6.6 | 90 | 121 | 2.3 |
| sole 2012 Scheldt (12/S/NL/S/004) n = 2 | mean | 19 | (9) | 57 | 85 | 0.98 |
|  | stand.dev. | 9 | 6 | 29 | 44 |  |
| sole 2013 Scheldt (13/S/NL/S/001) n = 2 | mean | (*1.1*) | (*0.7*) | (3.7) | (5.6) | 0.99 |
|  | stand.dev. | 1.6 | 1.0 | 5.3 | 5.8 |  |

n: number of replicate measurements or number of individually analyzed fish (2007).

Table S6: Details for the SPM samples (sampling for four periods of three months for each sampling campaign).

| **Year/Month** | **Color*** | **CO_3_^2-^ §** | **H_2_S $** | **Texture #** | **Odor** | **Amount of SPM (g ww)** |
| --- | --- | --- | --- | --- | --- | --- |
| **Rhône - Arles - France** | | | | | | |
| 2008-02 | 2.5y-3-3 | ++ | + | U, fs-ms | moldy/slightly fecal | 2963 |
| 2008-05 | 2.5y-3-4 | ++ | (+) | U, (fs)-ms | fecal | 1925 |
| 2008-08 | 2.5y-3-3 | ++ | + | U, fs, gs | fresh | 2523 |
| 2008-11 | 5y-4-4 | ++ | + | U, fs | fresh | 1069 |
|  |  |  |  |  |  | **sum: 8480 g ww** |
| 2010-02 | 2.5y 3-2 | +++ | + | fS, U | moldy | 1696 |
| 2010-05 | 2.5y 3-2 | +++ | - | fS, U | slightly moldy | 1814 |
| 2010-08 | 2.5y 3-4 | +++ | + | U, fS | moldy | 2105 |
| 2010-11 | 5y 4-2 | +++ | + | U, (fS) | moldy | 1248 |
|  |  |  |  |  |  | **sum: 6863 g ww** |
| 2012-02 | 2,5y-4-2 | +++ | ++ | fS, u | moldy | 5116 |
| 2012-05 | 2,5y-2-4 | +++ | (+) | fS, u | fishy | 2696 |
| 2012-08 | 5y-3-4 | ++ | + | fS, u | moldy | 2224 |
| 2012-11 | 2,5y-2-4 | ++ | (+) | U, fs (ms) | moldy | >5000 |
|  |  |  |  |  |  | **sum: > 15000 g ww** |
| 2014-02 | 2,5y-4-2 | +++ | + | fS,u | moldy | 4211 |
| 2014-05 | 2,5y-2-4 | ++ | - | fS,u | moldy | 1546 |
| 2014-08 | 2,5y-4-4 | ++ | - | fS,u | slightly moldy | 281 |
| 2014-11 | 2,5y-5-2 | ++ | - | U,fs (ms) | slightly moldy | 545 |
|  |  |  |  |  |  | **sum: 6583 g ww** |
| **Western Scheldt - Hansweert -NL** | | | | | | |
| 2008-02 | 2.5y-3-3 |  |  | sample was lost | | **-** |
| 2008-05**¥** | 2.5y-3,4 | + | - | U, fS-mS | fresh | 2165 |
| 2008-08 | 2.5y-3-2 |  |  | sample was lost | | **-** |
| 2008-11 | 5y-4-6 | + | + | U, fs | fresh | 890 |
|  |  |  |  |  |  | **sum: 3055 g ww** |
| 2010-02 | 2.5y 5-2 | + | - | U (fS) | moldy | 1832 |
| 2010-05 | 2.5y 3-2 | + | - | U (fS) | moldy | 1336 |
| 2010-08 | 2.5y 3-6 | ++ | - | U (fS) | moldy | 1561 |
| 2010-11 | 2.5y 3-6 | ++ | - | U, (fS), (mS) | fishy/moldy | 1280 |
|  |  |  |  |  |  | **sum: 6009 g ww** |
| 2012-02 | 2,5y-4-4 | ++ | - | U (fs) | fishy | 3499 |
| 2012-05 | 2,5y-3-6 | ++ | + | U, fs | moldy | 2617 |
| 2012-08 | 2,5y-4-10 | + | - | U, fs | moldy | 1520 |
| 2012-11 | 2,5y-5-2 | ++ | - | U, fs | fishy | 3808 |
|  |  |  |  |  |  | **sum: 11444 g ww** |
| 2014-02 | 2,5y-4-4 | +++ | - | U(fs) | fishy-moldy | 4000 |
| 2014-05 | 2,5y-3-2 | ++ | ++ | U,fs | moldy | 1089 |
| 2014-08 | 2,5y-4-4 | ++ | - | U,fs | fishy-moldy | 1138 |
| 2014-11 | 2,5y-5-2 | ++ | - | U,fs | slightly fishy | 1424 |
|  |  |  |  |  |  | **sum: 7651 g ww** |
| **Tees - Stockton - UK** | | | | | | |
| 2008-02 | 2.5y-4-2 | - | (+) | U, fs | slightly fecal | 3116 |
| 2008-05 | 2.5y-4-3 | - | (+) | U, (fs) | slightly earthy | 1220 |
| 2008-08 | 2.5y-4-3 | - | (+) | U, fs | fresh | 1785 |
| 2008-11 | 5y-3-6 | - | + | U, fs | fresh | 1267 |
|  |  |  |  |  |  | **sum: 7388 g ww** |
| 2010-02 | 2.5y 4-6 | - | + | U, (fS) | moldy | 1526 |
| 2010-05 | 2.5y 3-4 | - | - | U | moldy | 546 |
| 2010-08 | 2.5y 4-2 | - | - | U, (fS) | moldy | 960 |
| 2010-11 | 2.5y 4-3 | - | - | U, (fS) | moldy | 1135 |
|  |  |  |  |  |  | **sum: 4167 g ww** |
|  |  |  |  |  |  |  |
| 2012-02 | 2,5y-3-4 | - | + | U (fs) | moldy | 2833 |
| 2012-05 | 2,5y-3-4 | - | - | U, fs | fishy/moldy | 1884 |
| 2012-08 | 2,5y-3-6 | - | - | U | moldy | 2369 |
| 2012-11**∆** | 2,5y-4-4 | - | - | U (fs) | moldy | 3943 |
|  |  |  |  |  |  | **sum: 11029 g ww** |
| 2014-02 | 2,5y-3-2 | - | + | U(fs) | moldy | 3178 |
| 2014-05 | 2,5y-2-4 | - | + | U,fs | moldy | 532 |
| 2014-08 | 2,5y-3-4 | - | + | U | moldy | 401 |
| 2014-11 | 5y-3-2 | - | - | U | slightly moldy | 659 |
|  |  |  |  |  |  | **sum: 4770 g ww** |

* The code of the color is according to the Munsell Soil Color Chart. # Texture abbreviations:
S sand, U silt, f fine; if the symbol is in brackets it is only a minor fraction. § carbonate content: - no CO_3_^2-^;
+ low; ++ medium; +++ high; $ sulfide smell: - no; + yes; ¥ sampling site had to be changed after May 2008 (current was too high). ∆ severe flood event at Tees before sampling in November 2012.

Table S7: Concentrations of α-, β- and γ-HBCD in bi-annually sampled surface sediment (Lake Belau) and SPM at five European river sites. **Data are given as µg kg^-1^ dry weight.** For data below LOQ (LOD) measured values are reported and used for ΣHBCD and mean value calculations. Data below LOQ are in parentheses (data below LOD *in italics*).

| **site** | | | | | | | **α-HBCD** | **β-HBCD** | **γ-HBCD** | **ΣHBCD** |
| --- | --- | --- | --- | --- | --- | --- | --- | --- | --- | --- |
|  |  | | | |  | | **[µg kg^-1^ dw]** | **[µg kg^-1^ dw]** | **[µg kg^-1^ dw]** | **[µg kg^-1^ dw]** |
| **sediment samples (upper 2-3 cm layer from 16 cores; each sample = 4 cores)** | | | | | | | | | |  |
| Lake Belau cores 1 0-2 cm 12/08 | | | | | | | **(0.4)** | **(0.6)** | 1.0 | 2.0 |
| Lake Belau cores 2 0-2 cm 12/08 | | | | | | | 2.1 | 2.5 | 2.5 | 7.0 |
| Lake Belau cores 3 0-2 cm 12/08 | | | | | | | **(0.5)** | 2.0 | 1.7 | 4.1 |
| Lake Belau cores 4 0-2 cm 12/08 | | | | | | | **0.2** | **(0.3)** | 1.0 | 1.5 |
| **Lake Belau cores 0-2 cm 12/08 (n = 4)** | | | | | | **mean** | **(0.8)** | **1.3** | **1.5** | **3.7** |
|  |  | | | | **std.dev.** | | 0.9 | 1.1 | 0.7 | 2.5 |
|  | | | | | | |  |  |  |  |
| Lake Belau cores 1 0-2 cm 01/11 | | | | | | | 2.7 | 1.7 | 5.4 | 9.8 |
| Lake Belau cores 2 0-2 cm 01/11 | | | | | | | 2.8 | 1.4 | 2.9 | 7.1 |
| Lake Belau cores 3 0-2 cm 01/11 | | | | | | | 5.0 | 3.3 | 15.2 | 23.5 |
| Lake Belau cores 4 0-2 cm 01/11 | | | | | | | 2.1 | 1.0 | 2.1 | 5.2 |
| **Lake Belau cores 0-2 cm 01/11 (n = 4)** | | | | | | **mean** | **3.2** | **1.9** | **6.4** | **11.4** |
|  |  | | | | **std.dev.** | | 1.3 | 1.0 | 6.0 | 8.3 |
|  |  | | | |  | |  |  |  |  |
| Lake Belau cores 1 0-2 cm 12/12 | | | | | | | **(0.9)** | 1.3 | 4.8 | 7.1 |
| Lake Belau cores 2 0-2 cm 12/12 | | | | | | | 1.4 | 2.3 | 4.7 | 8.3 |
| Lake Belau cores 3 0-2 cm 12/12 | | | | | | | 3.7 | 1.5 | 4.9 | 10.1 |
| Lake Belau cores 4 0-2 cm 12/12 | | | | | | | 13.7 | 12.2 | 44.5 | 70.4 |
| **Lake Belau cores 0-2 cm 12/12 (n = 4)** | | | | | **mean** | | **4.9** | **4.3** | **14.7** | **24.0** |
|  |  | | | | **std.dev.** | | 6.0 | 5.2 | 19.9 | 31.0 |
|  |  | | | |  | |  |  |  |  |
|  |  | | | |  | | **05.02.2015** |  |  |  |
| Lake Belau cores 1 0-2 cm 11/14 | | | | | | | **(0.7)** | **(0.6)** | 2.8 | 4.1 |
| Lake Belau cores 2 0-2 cm 11/14 | | | | | | | **(0.1)** | **(0.6)** | 5.1 | 5.8 |
| Lake Belau cores 4 0-2 cm 11/14 | | | | | | | **(0.0)** | **(0.8)** | 7.6 | 8.4 |
| **Lake Belau cores 0-2 cm 11/14 (n = 3)** | | | | | **mean** | | **(0.3)** | **(0.7)** | **5.2** | **6.1** |
|  |  | | | | **std.dev.** | | 0.4 | 0.1 | 2.4 | 2.2 |
|  |  | | | |  | |  |  |  |  |
| **SPM samples (from passive sampling with traps)** | | | | | | | | | | |
|  |  | | | |  | |  |  |  |  |
| SPM 2008 Rhône | | | | | | | 18 | 3.6 | 19 | 41 |
| SPM 2008 Rhône | | | | | | | 50 | 9.4 | 10 | 69 |
| **SPM 2008 Rhône (n=2)** | | | | | **mean** | | **34** | **6.5** | **15** | **55** |
|  |  | | | | **std.dev.** | | 23 | 4 | 7 | 20 |
|  |  | | | | **variation** | | 16 | 3 | 5 | 14 |
|  |  | | | |  | |  |  |  |  |
| SPM 2010 Rhône (Feb) | | | | | | | 1.6 | **(0.3)** | 3.1 | 5.1 |
| *SPM 2010 Rhône (May)* | | | | | *outlier* | | *#36.0* | *#6.45* | *#5.87* | *#48.3* |
| *SPM 2010 Rhône (May)* | | | | | | | ***(0.7)*** | ***(0.2)*** | ***(0.7)*** | *1.6* |
| *SPM 2010 Rhône (May)* | | | | | | | ***(0.7)*** | ***(0.2)*** | ***(0.9)*** | *1.7* |
| *SPM 2010 Rhône (May)* | | | | | | | ***(0.8)*** | ***(0.)3*** | *4.1* | *5.2* |
| SPM 2010 Rhône (May) | | | mean May (n = 3) | | | | **(0.7)** | **(0.2)** | 1.9 | 2.8 |
| SPM 2010 Rhône (Aug) | | | | | | | 1.7 | **(0.3)** | **(0.6)** | 2.7 |
| *SPM 2010 Rhône (Nov)* | | | | | | | *1.9* | ***(0.4)*** | *2.3* | *4.6* |
| *SPM 2010 Rhône (Nov)* | | | | | | | *2.9* | ***(0.6)*** | *2.9* | *6.4* |
| SPM 2010 Rhône (Nov) | | | | mean Nov. (n = 2) | | | 2.4 | **(0.5)** | 2.6 | 5.5 |
| **SPM 2010 Rhône** | | **mean 2010 (n = 4)** | | | | | **1.6** | **(0.3)** | **2.1** | **4.0** |
|  |  | | | | **std.dev.** | | 0.7 | 0.1 | 1.1 | 1.5 |
|  |  | | | |  | |  |  |  |  |
| SPM 2012 Rhône | | | | | | | 1.4 | **(0.4)** | 1.8 | 3.5 |
| SPM 2012 Rhône | | | | | | | 5.2 | 1.0 | 2.0 | 8.2 |
| **SPM 2012 Rhône (n=2)** | | | | | **mean** | | **3.3** | **(0.7)** | **1.9** | **5.9** |
|  |  | | | | **std.dev.** | | 2.7 | 0.5 | 0.1 | 3.3 |
|  |  | | | |  | |  |  |  |  |
| SPM 2014 Rhône | | | | | | | **(0.9)** | **(0.3)** | 11.3 | 12.4 |
| SPM 2014 Rhône | | | | | | | 1.0 | **(0.4)** | 11.6 | 12.9 |
| **SPM 2014 Rhône (n=2)** | | | | | | **mean** | **(0.9)** | **(0.3)** | **11.4** | **12.7** |
|  |  | | | | **std.dev.** | | 0.1 | 0.1 | 0.2 | 0.4 |
|  |  | | | |  | |  |  |  |  |
| SPM 2008 Schelde | | | | | # outlier | | #137 | #26 | #54 | #216 |
| SPM 2008 Schelde | | | | | | | 2.6 | (0.9) | 16.9 | 20.4 |
| SPM 2008 Schelde | | | | | # outlier | | #2.3 | #(0.9) | #97 | #100 |
| SPM 2008 Schelde | | | | | | | 6.1 | 1.4 | 22.1 | 29.6 |
| SPM 2008 Schelde | | | | | | | 2.1 | (0.7) | 25.2 | 27.9 |
| SPM 2008 Schelde | | | | | | | 6.4 | 1.7 | 32.3 | 40.4 |
| SPM 2008 Schelde | | | | | # outlier | | #13 | #3.3 | #27 | #43 |
| SPM 2008 Schelde | | | | | | | 3.1 | (0.9) | 33.8 | 37.8 |
| SPM 2008 Schelde | | | | | | | 4.3 | 1.4 | 37.5 | 43.2 |
| **SPM 2008 Schelde (n=6)** | | | | | | **mean** | **4.1** | **1.2** | **27.9** | **33.2** |
|  |  | | | | **std.dev.** | | 1.8 | 0.4 | 7.8 | 8.7 |
|  |  | | | |  | |  |  |  |  |
| SPM 2010 Schelde | | | | | | | 3.1 | **(0.8)** | 44 | 47 |
| SPM 2010 Schelde | | | | | | | 4.8 | 1.2 | 23 | 29 |
| **SPM 2010 Schelde (n=2)** | | | | | | **mean** | **4.0** | **1.0** | **33** | **38** |
|  |  | | | | **std.dev.** | | 1.2 | 0.3 | 14.8 | 13.4 |
|  |  | | | |  | |  |  |  |  |
| SPM 2012 Schelde | | | | | | | 2.1 | **(0.7)** | 15 | 18 |
| SPM 2012 Schelde | | | | | | | 2.8 | **(0.9)** | 26 | 30 |
| **SPM 2012 Schelde (n=2)** | | | | | | **mean** | **2.5** | **(0.8)** | **21** | **24** |
|  |  | | | | **std.dev.** | | 0.5 | 0.1 | 7.9 | 8.6 |
|  |  | | | |  | |  |  |  |  |
| SPM 2014 Schelde | | | | | | | 1.5 | **(0.7)** | 20 | 22 |
| SPM 2014 Schelde | | | | | | | 3.1 | **1.3** | 21 | 26 |
| **SPM 2014 Schelde (n=2)** | | | | | | **mean** | **2.3** | **(1.0)** | **21** | **24** |
|  |  | | | | **std.dev.** | | 1.2 | 0.4 | 1.3 | 2.8 |
|  |  | | | |  | |  |  |  |  |
| SPM 2008 Tees (Feb) | | | | | | | 121 | 29 | 182 | 331 |
| SPM 2008 Tees (May) | | | | | | | 60 | 20 | 114 | 194 |
| SPM 2008 Tees (Sept) | | | | | | | 40 | 8.7 | 104 | 152 |
| SPM 2008 Tees (Nov) | | | | | | | 25 | 7.3 | 71 | 104 |
| **SPM 2008 Tees (n = 4)** | | | | | | **mean** | **61** | **16** | **118** | **195** |
|  |  | | | | **std.dev.** | | 42 | 10 | 46 | 98 |
|  |  | | | |  | |  |  |  |  |
| SPM 2010 Tees | | | | | | | 113 | 23 | 92 | 227 |
| SPM 2010 Tees | | | | | | | 84 | 24 | 235 | 344 |
| **SPM 2010 Tees (n = 2)** | | | | | | **mean** | **99** | **24** | **164** | **286** |
|  |  | | | | **std.dev.** | | 20.0 | 1.2 | 101.1 | 82.4 |
|  |  | | | |  | |  |  |  |  |
| SPM 2012 Tees | | | | | | | 48 | 15 | 318 | 381 |
| SPM 2012 Tees | | | | | | | 39 | 23 | 236 | 298 |
| **SPM 2012 Tees (n = 2)** | | | | | | **mean** | **43** | **19** | **277** | **339** |
|  |  | | | | **std.dev.** | | 6 | 6 | 58 | 58 |
|  |  | | | |  | |  |  |  |  |
| SPM 2014 Tees | | | | | | | 43 | 11 | 213 | 267 |
| SPM 2014 Tees | | | | | | | 40 | 14 | 202 | 256 |
| **SPM 2014 Tees (n = 2)** | | | | | | **mean** | **42** | **13** | **208** | **262** |
|  |  | | | | **std.dev.** | | 2 | 3 | 8 | 8 |
|  |  | | | |  | |  |  |  |  |
| **SPM 2008 Götaälv** | | | | | | | **4.2** | **(0.8)** | **2.5** | **7.5** |
|  |  | | | |  | |  |  |  |  |
| **SPM 2008 Mersey** | | | | | | | **147** | **36.2** | **1144** | **1328** |
| # The Scheldt 2008 sample was used as laboratory reference material and analyzed 9 times during the project period; finally 3 outliers were eliminated | | | | | | | | | | |

Table S8: Concentrations of α-, β- and γ-HBCD in annual composite SPM samples (rivers) and sediment core samples (Lake Belau) from 2008, 2010, 2012 and 2014 as **TOC-normalized data [µg kg^-1^ TOC].** Data below the LOQ are given in brackets.

| **year** | **α-HBCD** | **β-HBCD** | **γ-HBCD** | **ΣHBCD** | **TOC content** |  |
| --- | --- | --- | --- | --- | --- | --- |
|  | **[µg kg^-1^ TOC]** | **[µg kg^-1^ TOC]** | **[µg kg^-1^ TOC]** | **[µg kg^-1^ TOC]** | **[%]** |  |
|  | **sediment samples (upper 2 cm layer from core)** | | | |  |  |
|  | Lake Belau (n = 3 - 4) | | | |  |  |
| 2008 | (8 + 8) | 13 + 10 | 15 + 6 | 36 + 23 | 10.0 + 0.9 |  |
| 2010 | 31 + 11 | 18 + 9 | 63 + 57 | 113 + 77 | 9.9 + 0.7 |  |
| 2012 | 43 + 52 | 38 + 46 | 130 + 173 | 212 + 270 | 11.1 + 0.5 |  |
| 2014 | (3 + 4) | (7 + 1) | 54 + 26 | 63 + 23 | 9.7 + 0.2 |  |
|  | **SPM samples (from passive sampling with traps)** | | | |  |  |
|  | Rhône (n = 2 - 4) | | | |  |  |
| 2008 | 660 + 442 | 126 + 80 | 287 + 128 | 1070 + 390 | 5.1# |  |
| 2010 | 90 + 36$ | (18 + 5)$ | 111 + 64$ | 219 + 70$ | 1.9 + 0.7 |  |
| 2012 | 154 + 128 | (32 + 22) | 91 + 5 | 277 + 156 | 2.1# |  |
| 2014 | (44 + 5) | (15 + 4) | 539 + 9 | 598 + 17 | 2.1 + 0.1 |  |
|  | Western Scheldt (n = 2 - 6) | | | |  |  |
| 2008 | 158 + 71§ | 45 + 15§ | 1080 + 300§ | 1280 + 330§ | 2.6# |  |
| 2010 | 134 + 41 | 34 + 9 | 1120 + 501 | 1290 + 450 | 3.0# |  |
| 2012 | 79 + 16 | (24 + 5) | 659 + 251 | 761 + 272 | 3.2# |  |
| 2014 | 106 + 53 | (46 + 16) | 944 + 57 | 1100 + 130 | 2.2 + 0.2 |  |
|  | Tees (n = 2 - 4) | | | |  |  |
| 2008 | 737 + 558$ | 194 + 136$ | 1390 + 660$ | 2320 + 1350$ | 8.7 + 0.8 |  |
| 2010 | 1360 + 270 | 325 + 17 | 2250 + 1390 | 3930 + 1130 | 7.3# |  |
| 2012 | 605 + 87 | 264 + 80 | 3880 + 810 | 4750 + 820 | 7.2# |  |
| 2014 | 641 + 36 | 195 + 39 | 3210 + 120 | 4040 + 120 | 6.5 + 0.1 |  |
|  | Götaälv (n = 1) | | | |  |  |
| 2008 | 141 | (28) | 83 | 252 | 3.0# |  |
|  | Mersey (n = 1) | | | |  |  |
| 2008 | 1800 | 443 | 14000 | 16200 | 8.2# |  |

n - number of replicate measurements; # for TOC analysis n = 1; § The Scheldt 2008 sample was used as laboratory reference material and analyzed nine times during the project period of which three outliers were eliminated. $ Mean of four 3-months samples.

Table S9: Tees sediment samples 2013, upstream and downstream the barrage.

|  | **α-HBCD** | **β-HBCD** | **γ-HBCD** | **ΣHBCD** | **TOC** |
| --- | --- | --- | --- | --- | --- |
|  | **[µg kg^-1^ TOC]** | **[µg kg^-1^ TOC]** | **[µg kg^-1^ TOC]** | **[µg kg^-1^ TOC]** | **[g kg^-1^]** |
| **upstream samples (n = 4)** | | | | | |
| mean | 487 | 338 | 2980 | 3810 | 46 |
| std.dev. | 390 | 325 | 2820 | 3320 | 21 |
| no. | 4 | 4 | 4 | 4 | 4 |
| **downstream samples (n = 5)** | | | | | |
| mean | 232 | 135 | 3620 | 3990 | 54 |
| std.dev. | 185 | 119 | 2440 | 2720 | 9 |
| no. | 5 | 5 | 5 | 5 | 5 |

n - number of samples.

Table S10: Biota-sediment accumulation factors (BSAFs) and biota-suspended solids accumulation factors (BSSAFs) for ΣHBCD calculated as c_fish_ [µg kg^-1^ lipid weight] / c_solid_ [µg kg^-1^ TOC]. Lipid-normalized fish muscle ΣHBCD concentrations are taken from Tables S3/S5 and TOC-normalized sediment/SPM ΣHBCD data from Table S8.

| **site /year** | **ΣHBCD fish** | **ΣHBCD sediment/SPM** | **BSAF / BSSAF** |
| --- | --- | --- | --- |
|  | **[µg kg^-1^ lipid]** | **[µg kg^-1^ TOC]** | **(kg lipid) (kg TOC)^-1^** |
| **Lake Belau** | bream | sediment cores | BSAF |
| 2008 | 584 | 36 | 16.1 |
| 2010 | 84 | 113 | 0.74 |
| 2012 | 24 | 212 | 0.11 |
| **Götaälv** | bream | SPM | BSSAF |
| 2008 | 81 | 252 | 0.32 |
| **Tees** | bream | SPM | BSSAF |
| 2008 | 9482 | 2317 | 4.1 |
| 2010 | 11157 | 3930 | 2.8 |
| 2012 | 10096 | 4746 | 2.1 |
| **Rhône** | bream | SPM | BSSAF |
| 2008 | 1030 | 1073 | 1.0 |
| 2010 | 205 | 219 | 0.9 |
| 2012 | 299 | 277 | 1.1 |
| **Mersey** | bream | SPM | BSSAF |
| 2008 | 3139 | 16247 | 0.19 |
| **Western Scheldt** | **bream** | SPM | BSSAF |
| 2008 | 70 | 1282 | 0.05 |
| 2010 | 36 | 1285 | 0.03 |
| 2012 | 45 | 761 | 0.06 |
| **Western Scheldt** | **sole** | SPM | BSSAF |
| 2008 | 180 | 1282 | 0.14 |
| 2010 | 12 | 1285 | 0.01 |
| 2012 | 85 | 761 | 0.11 |


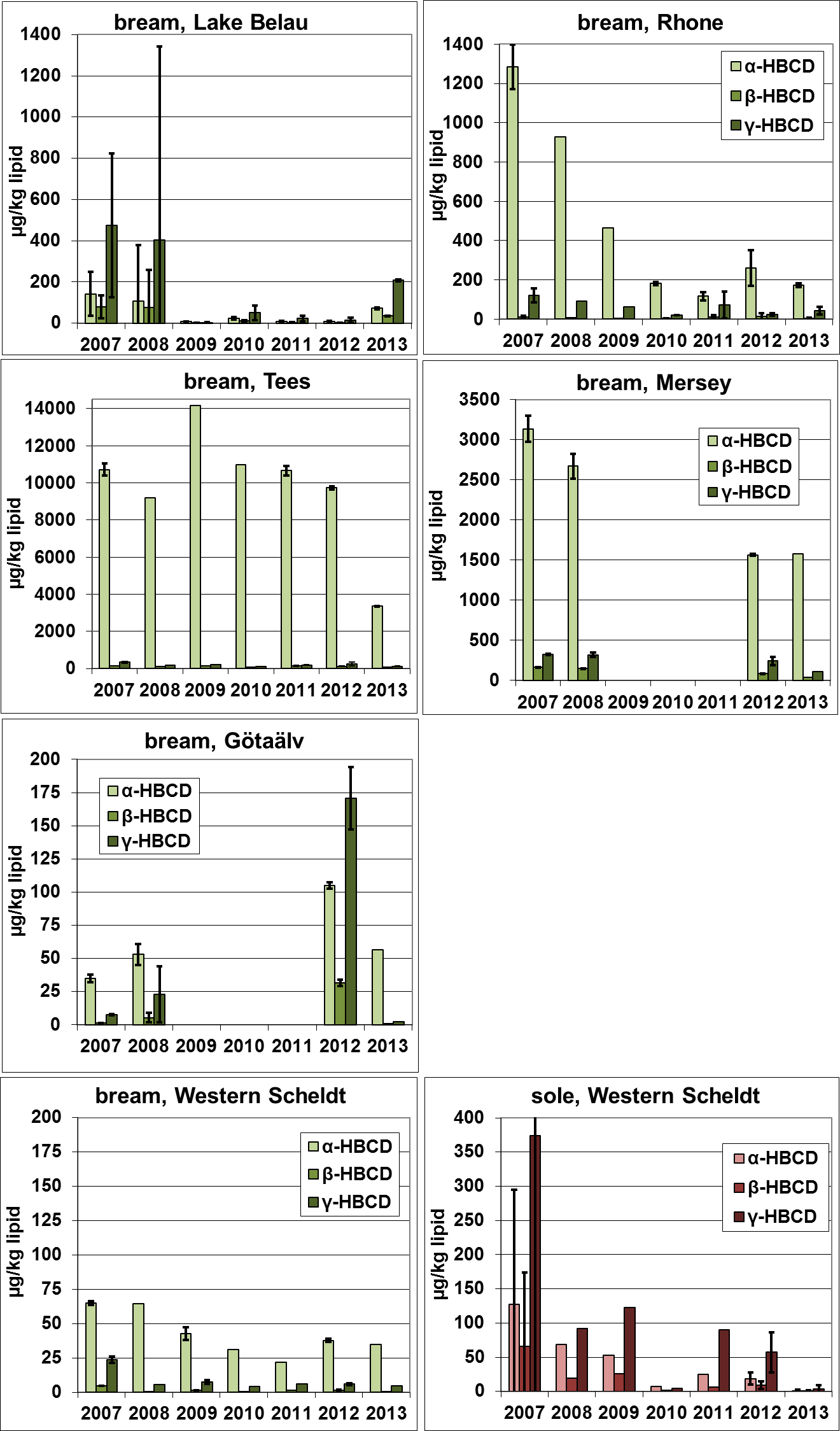


Fig. S1 Concentrations of α-, β-, and γ-HBCD in fish muscle tissue (**lipid weight data; µg kg^-1^ lipid**). The standard deviations are derived from replicate analyses of the same annual pool sample (n = 2 - 4; measure of analytical reproducibility) or from analyses of 15 individual fish (Lake Belau bream 2008; Western Scheldt sole 2007; measure of variability of individual fish burdens).


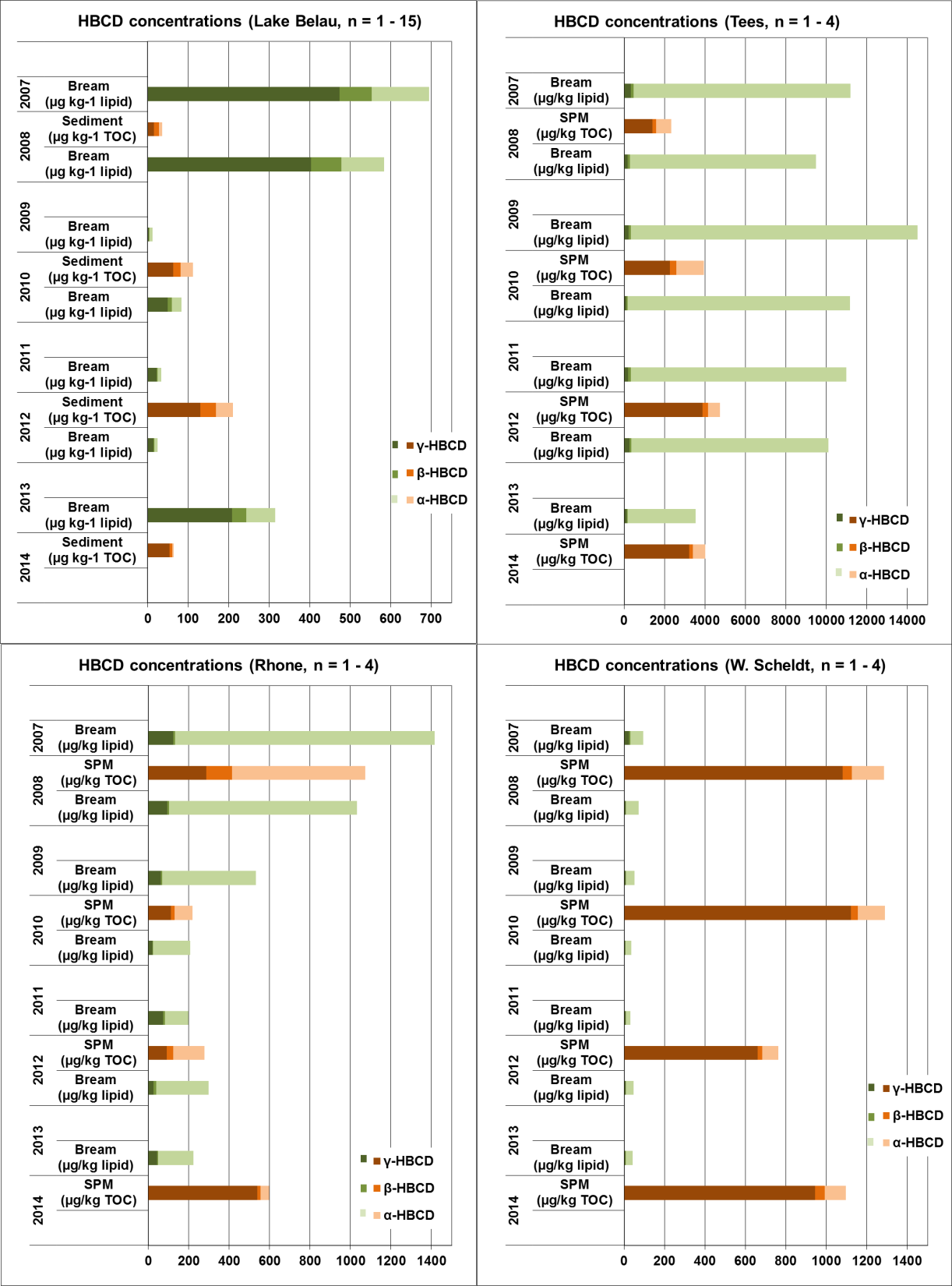


Fig. S2 Concentrations of α-, β-, and γ-HBCD in fish filet (green bars, lipid weight data; µg kg^-1^ lipid) and SPM/sediment (brown bars, TOC normalized data; µg kg^-1^ TOC). The data are mean values of replicate analyses (n = 2 - 4) of annual samples. Fish data are for pools of about 15 fish or mean values from 15 individually analyzed fish (Lake Belau bream 2008). SPM data are for pools of four 3-months SPM samples or mean values from four 3-months samples analyzed separately (Tees 2008 and Rhône 2010). Sediment data (Lake Belau) are for four samples prepared from four cores each.
